# Effectiveness and Safety of Apatinib Plus Chemotherapy as Neoadjuvant Treatment for Locally Advanced Gastric Cancer: A Nonrandomized Controlled Trial

**DOI:** 10.1001/jamanetworkopen.2021.16240

**Published:** 2021-07-09

**Authors:** Jian-Xian Lin, Yan-Chang Xu, Wei Lin, Fang-Qin Xue, Jian-Xin Ye, Wei-Dong Zang, Li-Sheng Cai, Jun You, Jian-Hua Xu, Jian-Chun Cai, Yi-Hui Tang, Jian-Wei Xie, Ping Li, Chao-Hui Zheng, Chang-Ming Huang

**Affiliations:** 1 Department of Gastric Surgery, Fujian Medical University Union Hospital, Fuzhou, Fujian Province, China; 2 Key Laboratory of Ministry of Education of Gastrointestinal Cancer, Fujian Medical University, Fuzhou, Fujian Province, China; 3 Department of Gastrointestinal Surgery, The First Hospital of Putian, Putian, Fujian Province, China; 4 Department of Gastrointestinal Surgery and Gastrointestinal Surgery Research Institute, The Affiliated Hospital of Putian University, Putian, Fujian Province, China; 5 Department of Gastrointestinal Surgery, Fujian Provincial Hospital, Fuzhou, Fujian Province, China; 6 Department of Gastrointestinal Surgery, The First Affiliated Hospital of Fujian Medical University, Fuzhou, Fujian Province, China; 7 Department of Gastrointestinal Surgery, Fujian Provincial Cancer Hospital, Fuzhou, Fujian Province, China; 8 Department of General Surgery, Zhangzhou Affiliated Hospital of Fujian Medical University, Zhangzhou, Fujian Province, China; 9 Department of Gastrointestinal Oncology Surgery, The First Affiliated Hospital of Xiamen University, Xiamen, Fujian Province, China; 10 Department of Oncology Surgery, The Second Affiliated Hospital of Fujian Medical University, Quanzhou, Fujian Province, China; 11 Department of Gastrointestinal Surgery, Zhongshan Hospital Affiliated to Xiamen University, Xiamen, Fujian Province, China

## Abstract

**Importance:**

Apatinib is a novel treatment option for chemotherapy-refractory advanced gastric cancer (GC), but it has not been evaluated in patients with locally advanced GC.

**Objective:**

To investigate the effectiveness and safety of apatinib combined with S-1 plus oxaliplatin (SOX) as a neoadjuvant treatment for locally advanced GC.

**Design, Setting, and Participants:**

This multicenter, prospective, single-group, open-label, phase 2 nonrandomized controlled trial was conducted in 10 centers in southern China. Patients with M0 and either clinical T2 to T4 or N+ disease were enrolled between July 1, 2017, and June 30, 2019. Statistical analysis was performed from December 1, 2019, to January 31, 2020.

**Interventions:**

Eligible patients received apatinib (500 mg orally once daily on days 1 to 21 and discontinued in the last cycle) plus SOX (S-1: 40-60 mg orally twice daily on days 1 to 14; oxaliplatin: 130 mg/m^2^ intravenously on day 1) every 3 weeks for 2 to 5 cycles. A D2 gastrectomy was performed 2 to 4 weeks after the last cycle.

**Main Outcomes and Measures:**

The primary end point was R0 resection rate. Secondary end points were the response rate, toxic effects, and surgical outcome.

**Results:**

A total of 48 patients (mean [SD] age, 63.2 [8.2] years; 37 men [77.1%]) were enrolled in this study. Forty patients underwent surgery (38 had gastrectomy, and 2 had exploratory laparotomy), with an R0 resection rate of 75.0% (95% CI, 60.4%-86.4%). The radiologic response rate was 75.0%, and T downstaging was observed in 16 of 44 patients (36.4%). The pathological response rate was 54.2% (95% CI, 39.2%-68.6%); moreover, this rate was significantly higher in patients who achieved a radiologic response compared with those who did not (12 [80.0%] vs 1 [20.0%]; *P* = .03) and in those who had an Eastern Cooperative Oncology Group Performance Status score of 0 (20 [76.9%] vs 10 [45.5%]; *P* = .03) or had tumors located in the upper one-third of the stomach (16 [61.5%] vs 7 [31.8%]; *P* = .04). Patients who achieved a pathological response (vs those who did not) had significantly less blood loss (median [range]: 60 [10-200] mL vs 80 [20-300] mL; *P* = .04) and significantly more lymph nodes harvested (median [range]: 40 [24-67] vs 32 [19-51]; *P* = .04) during surgery. Postoperative complications were observed in 7 of 38 patients (18.4%). Grade 3 toxic effects occurred in 16 of 48 patients (33.3%), and no grade 4 toxic effects or preoperative deaths were observed.

**Conclusions and Relevance:**

This nonrandomized controlled trial found that apatinib combined with SOX was effective and had an acceptable safety profile as a neoadjuvant treatment for locally advanced GC. A large-scale randomized clinical trial may be needed to confirm the findings.

**Trial Registration:**

ClinicalTrials.gov Identifier: NCT03192735

## Key Points

**Question:**

Is apatinib combined with S-1 plus oxaliplatin (SOX) effective and tolerable as a neoadjuvant treatment for locally advanced gastric cancer (GC)?

**Findings:**

In this nonrandomized controlled trial of 48 patients with locally advanced GC, those who received apatinib plus SOX had an R0 resection rate of 75.0% and a pathological response rate of 54.2%. This regimen also showed a good tolerability profile.

**Meaning:**

In this study, apatinib plus SOX was effective and had an acceptable safety profile for use as a neoadjuvant treatment in locally advanced GC; further studies are warranted to ascertain the effectiveness and safety of this regimen.

## Teaser

This nonrandomized controlled trial evaluates the resection rate, responses, survival, adverse events, and surgical outcomes associated with a drug regimen for locally advanced gastric cancer.

## Introduction

Worldwide, more than 1 million new cases and an estimated 783 000 deaths caused by gastric cancer were recorded in 2018, making it the fifth most frequently diagnosed cancer and the third-leading cause of cancer death.^1^ Endoscopic or surgical resection is curative in most early gastric cancers, with a 5-year overall survival (OS) rate that is greater than 90%.^2^ In contrast, prognosis remains poor for locally advanced gastric cancer (GC) even after the complete dissection of the primary tumors and regional lymph nodes.^3^ Thus, multimodal treatments have been proposed to prolong the survival period. Since the MAGIC trial first demonstrated in 2006 that perioperative chemotherapy (epirubicin hydrochloride, cisplatin, and fluorouracil) could increase the 5-year OS rate from 23% to 36% compared with surgery alone,^4^ systemic chemotherapy has become the standard treatment for locally advanced GC.^5^ In Asia, oral fluoropyrimidines (eg, S-1 or capecitabine) plus oxaliplatin is a first-line regimen.^6,7^ However, chemotherapy has been reported to be less effective against gastric cancer than against other solid malignant neoplasms because of the heterogeneity of tumors.^8,9^ Therefore, new, effective treatment options with acceptable safety profiles are urgently needed.

In the past decade, several clinical trials have investigated molecular targeted therapy for advanced GC, but few molecular agents have shown promising activity.^10,11,12,13^ The ToGA (Trastuzumab for Gastric Cancer) trial reported that the trastuzumab with cisplatin and capecitabine or fluorouracil regimen was associated with improved OS for *ERBB2* (formerly *HER2*)–positive advanced GC or gastroesophageal junction (GEJ) cancer.^10^ However, only a small percentage of patients (approximately 20%) are ideal candidates for *ERBB2* targeted therapy.^10,14,15^ Another well-established target is vascular endothelial growth factor (VEGF), which is one of the most potent angiogenic factors and a signaling molecule secreted by many solid tumors.^16,17^ Because a high level of VEGF expression is one of the characteristic features of gastric carcinomas, targeting VEGF is a potential therapeutic strategy. Apatinib, a novel receptor tyrosine kinase inhibitor that selectively targets VEGF receptor 2 (VEGFR2), has been shown to inhibit VEGF-mediated endothelial cell migration, proliferation, and tumor microvascular density.^18^ A phase 3 study showed that apatinib compared with placebo improved OS and progression-free survival in patients with chemotherapy-refractory advanced or metastatic adenocarcinoma of the stomach or GEJ.^19^

Neoadjuvant chemotherapy could decrease the tumor stage, increase the R0 resection rate, and provide survival benefits for patients with advanced GC.^20,21^ However, rare evidence supports the use of apatinib combined with chemotherapy in neoadjuvant treatment. Thus, we conducted the present nonrandomized controlled trial to investigate the effectiveness and safety of apatinib combined with S-1 plus oxaliplatin (SOX) as a neoadjuvant treatment for locally advanced GC.

## Methods

This multicenter, single-group, open-label, phase 2, nonrandomized controlled trial was undertaken in 10 centers in southern China (Fujian Medical University Union Hospital, Fuzhou; The First Hospital of Putian, Putian; The Affiliated Hospital of Putian University, Putian; Fujian Provincial Hospital, Fuzhou; The First Affiliated Hospital of Fujian Medical University, Fuzhou; Fujian Provincial Cancer Hospital, Fuzhou; Zhangzhou Affiliated Hospital of Fujian Medical University, Zhangzhou; The First Affiliated Hospital of Xiamen University, Xiamen; The Second Affiliated Hospital of Fujian Medical University, Quanzhou; and Zhongshan Hospital Affiliated to Xiamen University, Xiamen). Complete inclusion and exclusion criteria are listed in eTable 1 in Supplement 1. Briefly, patients aged 18 to 75 years with confirmed primary gastric adenocarcinoma (papillary, tubular, mucinous, signet ring cell, or poorly differentiated) without previous surgery, chemotherapy, radiotherapy, or targeted therapy and no distant metastases were included. Patients with M0 and either clinical T2 to T4 or N+ disease were enrolled between July 1, 2017, and June 30, 2019. Excluded patients included pregnant and/or lactating women; those with severe mental disorders; those with a history of chemotherapy, radiotherapy, or other malignant neoplasm within the past 5 years; those with unstable angina, myocardial infarction, or cerebrovascular accident in the past 6 months; and those with risk factors for gastrointestinal bleeding. The number of cases from each center is shown in eTable 2 in Supplement 1. All patients provided written informed consent before enrollment. The informed consent form and trial protocol (Supplement 2) were approved by the institutional review board at each participating center. The trial was conducted in accordance with the International Conference on Harmonization Good Clinical Practice guidelines, the Declaration of Helsinki,^22^ and Chinese law. We followed the Transparent Reporting of Evaluations With Nonrandomized Designs (TREND) reporting guideline.

### Treatment

All enrolled patients received 2 to 5 preoperative and 6 postoperative cycles of apatinib plus SOX every 3 weeks. This regimen consisted of the following: (1) oral administration of apatinib (courtesy of Jiangsu Hengrui Medicine), 500 mg, once daily on days 1 to 21 (only discontinued in the last cycle), (2) oral administration of S-1 at a dose based on body surface area (40 mg for body surface area <1.25 m^2^, 50 mg for 1.25 to 1.5 m^2^, and 60 mg for >1.5 m^2^) twice daily on days 1 to 14, and (3) intravenous infusion of oxaliplatin, 130 mg/m^2^, on day 1. In this trial, tumor response was first assessed before the initiation of the third course. Patients who achieved a good tumor response underwent surgery after completing treatment, whereas those with poor tumor response were given 2 more courses of neoadjuvant treatment and surgical candidacy was evaluated thereafter. Dose reductions were allowed in the presence of grade 3 hematologic or grade 2 nonhematologic toxic effects. For each drug, dose reductions were allowed up to 2 times. Treatment interruption was allowed for no more than 14 days (cumulatively) and no more than 2 times in each cycle. Dose reescalation was not permitted. The criteria for stopping treatment were disease progression or death, unbearable toxic effects after dose reductions, patient refusal to continue, and investigator decision that stopping treatment was in the best interest of the patient.

Safety and laboratory assessments, including vital signs, physical examination, weight, Eastern Cooperative Oncology Group performance status, laboratory tests, tumor markers, and 12-lead electrocardiograms, were conducted at baseline and before the start of every cycle. Adverse events (AEs) were recorded every week. Radiologic assessment by computed tomography (CT) or magnetic resonance imaging was provided to evaluate the tumor response.

Without clear surgical contraindications and with patient consent, surgery was performed 2 to 4 weeks after the completion of neoadjuvant treatment. Each center has an annual surgical volume greater than 150. All surgical procedures, including the extent of lymph node dissection, adhered to the guidelines of the Japanese Research Society for the Study of Gastric Cancer (eFigure 1 in Supplement 1),^23^ and staging was based on the TNM classification of the American Joint Committee on Cancer’s *Cancer Staging Manual*.^24^ Postoperative complications were assessed according to the Clavien-Dindo classification.^25^ All resected specimens were examined by a professional pathology team. Adjuvant treatment was started 3 to 8 weeks after gastrectomy.

### End Points and Assessments

The primary end point of this study was the R0 resection rate, defined as the proportion of patients with margin-free resection. Secondary end points included the response rate, toxic effects, and surgical outcome.

The radiologic response was assessed using contrast-enhanced CT or magnetic resonance imaging findings before and after neoadjuvant treatment according to the Response Evaluation Criteria in Solid Tumors (RECIST) guideline, version 1.1; response included complete response, partial response, stable disease, and progressive disease.^26^ For locally advanced GC, a measurable lesion was defined as a lymph node more than 15 mm in the short axis when assessed by CT scan (eFigure 2 in Supplement 1). Next, the radiologic response rate was calculated as the proportion of patients with partial response and complete response. The pathological response was evaluated using the modified Japanese Classification of Gastric Carcinoma, third English edition.^27^ The histologic response of the primary tumor was evaluated in the section in which the tumor was believed to have been located at the pretreatment assessment and in the sections in which tumor cells were likely to remain and included the following categories: grade 0, no degeneration; grade 1a, the degeneration area was less than one-third; grade 1b, the degeneration area was one-third to two-thirds; grade 2a, the degeneration area was two-thirds to nine-tenths; grade 2b, the degeneration area was more than nine-tenths; and grade 3, no residual tumor (eFigure 3 in Supplement 1). According to previous studies, the major pathological response rate was calculated as the proportion of patients with tumor regression that was greater than 90%, which corresponded to grade 2b and grade 3.^28^

The radiologic or pathological response was assessed by radiologists or pathologists with at least 5-year working experiences from each center, all of whom were blinded to this study. Toxic effects were evaluated according to the Common Terminology Criteria for Adverse Events, version 4.0.

### Statistical Analysis

The sample size calculation was based on the assumption that the R0 resection rate would be 75% with SOX alone and 90% with apatinib plus SOX. With a 1-sided α of 5% and a power of 80%, a sample size of 50 patients was calculated, including a 15% to 20% dropout rate. The full analysis set included patients who had received at least 1 cycle of neoadjuvant treatment. The per-protocol set included those who had completed neoadjuvant treatment and undergone surgery. Categorical variables were assessed using the χ^2^ test or Fisher exact test, and continuous variables were compared using an unpaired, 2-tailed *t* test or the Mann-Whitney test.

All statistical analyses were conducted with SPSS software, version 18.0 (IBM). A 2-sided *P* < .05 was considered to be statistically significant. Statistical analysis was performed from December 1, 2019, to January 31, 2020.

## Results

A total of 48 patients were enrolled in this study, excluding 2 patients who withdrew their consent to participate. Of these patients, 37 (77.1%) were men and 11 (22.9%) were women, and the mean (SD) age was 63.2 (8.2) years. Patients’ characteristics are shown in Table 1, and the flow diagram is provided in the Figure. All enrolled patients completed 156 preoperative cycles of apatinib plus SOX, with a median (range) of 3 (1-5) cycles. Seven of 48 patients (14.6%) discontinued preoperative treatment: 4 had disease progression, 2 experienced AEs, and 1 refused to continue the treatment. Of the remaining 41 patients (85.4%), 38 underwent gastrectomy, 2 underwent exploratory laparotomy because of peritoneal metastasis, and 1 refused surgery. Thirty of 38 patients (78.9%) received adjuvant treatment for 89 cycles after gastrectomy, with a median (range) of 3 (1-6) cycles.

**Table 1.** Baseline Demographic and Clinical Characteristics

Characteristic	No. (%)
Total patients, No.	48
Age, mean (SD), y	63.2 (8.2)
Sex	
Male	37 (77.1)
Female	11 (22.9)
Body weight index, median (range)^a^	22 (15.1-30.9)
ECOG PS score	
0	31 (64.6)
1/2	17 (35.4)
Tumor differentiation	
Well or moderately differentiated	12 (25.0)
Poorly differentiated or mucinous or signet ring cell carcinoma	25 (52.1)
Unknown	11 (22.9)
Lauren classification	
Intestinal	11 (22.9)
Diffuse	26 (54.2)
Unknown	11 (22.9)
Tumor location in the stomach	
Lower one-third	11 (22.9)
Middle one-third	14 (30.2)
Upper one-third	23 (47.9)
Borrmann type	
I/II	3 (6.3)
III	42 (87.5)
IV	3 (6.3)
cT stage	
T3	5 (10.4)
T4	43 (89.6)

Abbreviation: ECOG PS, Eastern Cooperative Oncology Group Performance Status.

^a^ Body mass index is calculated as weight in kilograms divided by height in meters squared.

**Figure.**
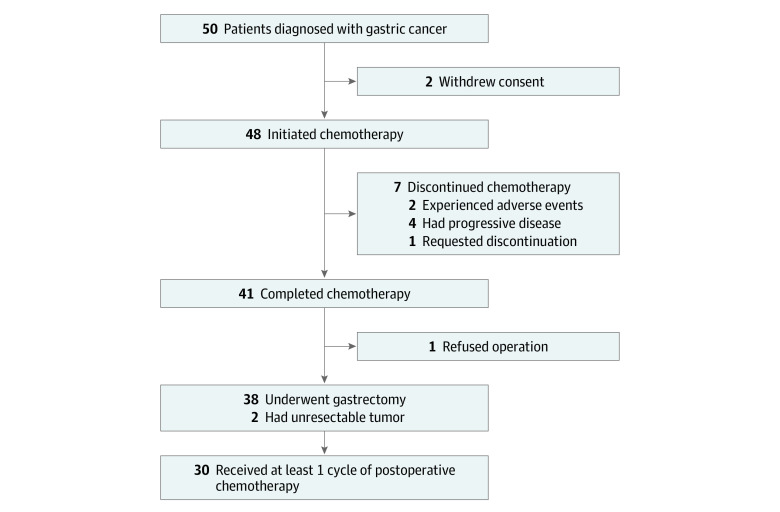
COHORT Diagram of Study Population

### Effectiveness

In the full analysis set, 36 of 48 patients underwent radical gastrectomy, with an R0 resection rate of 75.0% (36 of 48 patients; 95% CI, 60.4%-86.4%). In the per-protocol set, the R0 resection rate was 90.0% (36 of 40 patients; 95% CI, 76.3%-97.2%).

Forty-four patients (91.7%) were eligible for radiologic assessment, and 4 patients (8.3%) did not receive CT scans because they stopped treatment after 1 cycle. A total of 20 patients (45.5%) had target lesions. Among them, 1 patient (5.0%) had complete response, 14 (70.0%) had partial response, 4 (20.0%) had stable disease, and 1 (5.0%) had progressive disease, and the radiologic response rate was 75.0%. Of the 24 patients without target lesions, 3 (12.5%) had progressive disease and none had complete response. Among patients who had progressive disease, 1 (33.3%) experienced progression in the baseline lymph nodal lesions, 2 (66.7%) had new target lymph nodes, and 1 (33.3%) had new metastatic liver lesions. In a comparison of the pretreatment and the posttreatment CT staging, T downstaging occurred in 16 of 44 patients (36.4%), including 10 patients (62.5%) with downstaging from T4 to T3, 4 patients (25.0%) with T4 to T2, and 2 patients (12.5%) with T4 to T1; N downstaging (N+ to N–) occurred in 4 of 41 patients (9.8%) (eTable 3 in Supplement 1).

The pathological responses of patients who proceeded to surgery are depicted in eFigure 4 in Supplement 1. Twenty-six patients had grade Ib or greater pathological response, with a pathological response rate of 54.2% (95% CI, 39.2%-68.6%). Moreover, the main pathological response was observed in 12 patients (25.0%; 95% CI, 13.6%-39.6%), and a pathological complete response was observed in 3 patients (6.3%; 95% CI, 1.3%-17.2%). The pathological response rate was 80% (12 of 15 patients) and the major pathological response rate was 33.3% (5 of 15 patients) among those who achieved a radiologic response, suggesting that the pathological response did not always agree with the radiologic response. eFigure 5 in Supplement 1 depicts the pathological response stratified by preoperative cycles in patients who underwent surgery. Most pathological responses and major pathological responses were achieved in patients who completed 3 cycles, suggesting that this time point may be ideal for evaluating the effectiveness of treatment.

### Surgical Outcomes

Table 2 shows the surgical and pathological characteristics of the patients who underwent gastrectomy. All patients underwent D2 lymphadenectomy. Thirty-three patients (86.8%) received total gastrectomy, and 5 (13.2%) received distal gastrectomy. Table 3 details the surgical outcomes. The median (range) of lymph nodes harvested was 39 (19-67). The median (range) intraoperative blood loss was 100 (10-600) milliliters. The median (range) postoperative hospital stay was 10 (5-28) days. Seven of 38 patients (18.4%) experienced postoperative complications, including 4 (57.1%) patients with pneumonia, 1 (14.3%) with postoperative bleeding, 1 (14.3%) with abdominal infection, and 1 (14.3%) with ileus. All complications were categorized as Clavien-Dindo grade II. No readmission, reoperation, or postoperative death within 30 days was observed.

**Table 2.** Surgical Findings

Variable	Patients, No. (%) (N = 48)
Type of gastrectomy	
Total	33 (86.8)
Distal	5 (13.2)
Tumor differentiation	
Well or moderately differentiated	11 (28.9)
Poorly differentiated or mucinous or signet ring cell carcinoma	21 (55.3)
Unknown	6 (15.8)
Lauren classification	
Intestinal	10 (26.3)
Diffuse	22 (57.9)
Unknown	6 (15.8)
Lymphovascular invasion	
No	20 (52.6)
Yes	18 (47.4)
Neural invasion	
No	21 (55.3)
Yes	17 (44.7)
T stage	
T0	3 (7.9)
T1	4 (10.5)
T2	2 (5.3)
T3	15 (39.5)
T4a	12 (31.6)
T4b	2 (5.3)
N stage	
N0	12 (31.6)
N1	7 (18.4)
N2	7 (18.4)
N3a	6 (15.8)
N3b	6 (15.8)
R category	
R0	36 (94.7)
R1	2 (5.3)

**Table 3.** Surgical Outcomes

Variable	All	Tumor regression		*P* value
		<One-third	≥One-third	
No. of patients	38	12	26	NA
Bleeding, median (range), mL	100 (10-600)	80 (20-300)	60 (10-200)	.04
Lymph node harvested, median (range)	39 (19-67)	32 (19-51)	40 (24-67)	.04
Time to food intake, median (range), d	4 (2-10)	4 (2-10)	4 (2-8)	.35
Postoperative hospital stay, median (range), d	10 (5-28)	10 (6-18)	10 (5-28)	.51
Surgical complications, No. (%)				
Total	7 (18.4)	2 (16.7)	5 (19.2)	.62
Postoperative bleeding	1 (2.6)	1 (8.3)	0	
Abdominal infection	1 (2.6)	0	1 (3.8)	
Pneumonia	4 (10.5)	1 (8.3)	3 (11.5)	
Ileus	1 (2.6)	0	1 (3.8)	

Abbreviation: NA, not applicable.

### Association Between Pathological Responses and Patient Characteristics

To further identify the patients who were more likely to benefit from apatinib combined with SOX, we compared the baseline characteristics between the patients who achieved a pathological response and those who did not. The results showed that the pathological response rate was significantly higher in the patients with an Eastern Cooperative Oncology Group Performance Status score of 0 (20 [76.9%] vs 10 [45.5%]; *P* = .03) or tumors located in the upper one-third of the stomach (16 [61.5%] vs 7 [31.8%]; *P* = .04) (eFigure 6 and eTable 4 in Supplement 1). Moreover, patients who achieved a pathological response compared with those who did not had significantly less blood loss (median [range]: 60 [10-200] mL vs 80 [20-300] mL; *P* = .04) and significantly more lymph nodes harvested (median [range]: 40 [24-67] nodes vs 32 [19-51] nodes; *P* = .04) during the surgical procedure, suggesting that achieving a pathological response was associated with better surgical outcomes.

### Adverse Effects

The nonsurgical, preoperative AEs experienced by patients in the apatinib plus SOX group are listed in Table 4. The most common toxic effects were neutropenia (22 patients [45.8%]), followed by leukopenia (21 [43.8%]), increased transaminase (20 [41.7%]), and anemia (18 [37.5%]). All-grade hypertension occurred in 16 patients (33.3%), hand-foot syndrome in 10 (20.8%), and proteinuria in 9 (18.8%). A total of 16 patients (33.3%) experienced grade 3 AEs, and the most common (>1 case) were neutropenia (2 [4.2%]), thrombocytopenia (2 [4.2%]), anorexia (2 [4.2%]), and bleeding (2 [4.2%]). No grade 4 AEs or preoperative deaths were observed. The mean number of preoperative cycles was significantly lower in patients who experienced grade 3 AEs compared with those who did not (2.7 vs 3.5 cycles; *P* = .009), suggesting that the incidence of AEs was not associated with the cumulative exposure to the systemic drugs.

**Table 4.** Adverse Events Graded Using the Common Terminology Criteria for Adverse Events 4.0

Adverse event	Total No. (%) (N = 48)	Grade, No. (%)		
		1 (n = 41)	2 (n = 28)	3 (n = 16)
Hematologic				
Neutropenia	22 (45.8)	10 (20.8)	10 (20.8)	2 (4.2)
Leukopenia	21 (43.8)	16 (33.3)	5 (10.4)	0
Thrombocytopenia	17 (35.4)	7 (14.6)	8 (16.7)	2 (4.2)
Anemia	18 (37.5)	11 (22.9)	4 (8.3)	3 (6.3)
Nonhematologic				
Hand-foot syndrome	10 (20.8)	7 (14.6)	3 (6.3)	0
Hypertension	16 (33.3)	9 (18.8)	7 (14.6)	0
Proteinuria	9 (18.8)	6 (12.5)	2 (4.2)	1 (2.1)
Increased transaminase	20 (41.7)	17 (35.4)	2 (4.2)	1 (2.1)
Increased bilirubin	14 (29.2)	10 (20.8)	3 (6.3)	1 (2.1)
Nausea	11 (22.9)	5 (10.4)	5 (10.4)	1 (2.1)
Vomiting	7 (14.6)	3 (6.3)	3 (6.3)	1 (2.1)
Anorexia	9 (18.8)	3 (6.3)	4 (8.3)	2 (4.2)
Diarrhea	6 (12.5)	3 (6.3)	2 (4.2)	1 (2.1)
Fatigue	12 (25.0)	7 (14.6)	5 (10.4)	0
Pain	4 (8.3)	1 (2.1)	2 (4.2)	1 (2.1)
Bleeding	6 (12.5)	2 (4.2)	2 (4.2)	2 (4.2)

## Discussion

To our knowledge, this nonrandomized controlled trial is the first multicenter, prospective trial to explore the effectiveness and safety of apatinib plus chemotherapy as a neoadjuvant treatment for locally advanced GC worldwide. Between July 1, 2017, and June 30, 2019, we enrolled 48 patients from 10 centers who did not receive previous anticancer treatment. Thirty-six patients underwent radical gastrectomy, with an R0 resection rate of 75.0%. The pathological response rate was 54.2%, and the major pathological response rate was 25.0%. The toxic effects and surgical complications were manageable, and no drug-related or postoperative deaths were observed. These findings support the use of apatinib plus SOX as a neoadjuvant treatment for locally advanced GC.

Angiogenesis is universally considered a cancer hallmark, as it supplies the increased request for oxygen and nutrients, which is typical of the fast-growing microenvironment of solid tumors.^16^ As the main factor responsible for tumor angiogenesis, VEGFR2 represents an appealing target for novel anticancer therapies. Currently, it is possible to inhibit the signal arising from the activation of VEGFR2 through several pharmacodynamic approaches, including receptor blockade (ramucirumab), seizure of the ligand (bevacizumab), and small-molecule inhibition (sorafenib tosylate, sunitinib, and apatinib).^29^

Several studies have demonstrated the effectiveness of these drugs in gastric or GEJ carcinoma. Among these drugs, apatinib seems to be the most promising antiangiogenic agent. A randomized, double-blind, placebo-controlled phase 3 trial^19^ assessed the effectiveness and safety of apatinib in 267 patients from 32 centers who had advanced gastric or GEJ adenocarcinoma that was refractory to 2 or more lines of previous chemotherapy. Compared with placebo, apatinib was associated with significantly improved OS (6.5 vs 4.7 months; *P* = .02) and progression-free survival (2.6 vs 1.8 months; *P* < .001) with an acceptable safety profile. However, the ANGEL study, a global, randomized, placebo-controlled phase 3 trial, did not demonstrate an OS benefit for rivoceranib (apatinib) vs placebo (5.8 vs 5.1 months; *P* = .49) in patients with advanced GC or metastatic gastric cancer in Asia Pacific, North America, and Europe.^30^ Moreover, considering the limited benefit of apatinib monotherapy, the effectiveness of apatinib combined with chemotherapy should be investigated.^18^ In a single-group phase 2 trial involving 29 patients with locally advanced GC, apatinib combined with SOX and followed by surgery showed favorable activity and manageable safety.^31^ Because of the single-center design of the previous study, this present multicenter, prospective study was conducted to investigate the effectiveness and safety of apatinib plus SOX with the same dose in the more general population and to ascertain the optimal number of neoadjuvant treatment cycles.

Surgery is the cornerstone in gastric cancer treatment. Achieving R0 resection is considered the goal of surgery, especially for locally advanced GC.^32,33,34^ Therefore, the R0 resection rate was the primary end point of this study. As previously reported, preoperative chemotherapy was associated with increased R0 resection rate in patients with locally advanced GC. In the FNCLCC/FFCD 9703 study, 224 patients with resectable adenocarcinoma of the lower esophagus, GEJ, or stomach were randomly assigned to either perioperative chemotherapy plus surgery (n = 113) or surgery alone (n = 111).^35^ Patients in the chemotherapy plus surgery group had a significantly improved curative resection rate (84% vs 73%; *P* = .04), 5-year disease-free survival (34% vs 19%; *P* = .003), and 5-year OS (38% vs 24%; *P* = .02) compared with the surgery-alone group.^35^ Moreover, significant differences in the improvement of the R0 resection rate were observed among various treatment regimens. In the FLOT4-AIO study, 300 eligible patients were randomly assigned to either the epirubicin, cisplatin, and fluorouracil or epirubicin, cisplatin, and capecitabine group (n = 152) or the docetaxel, oxaliplatin, leucovorin, and fluorouracil group (n = 148).^36^ The R0 resection rate of the docetaxel, oxaliplatin, leucovorin, and fluorouracil group was significantly higher than that of the epirubicin, cisplatin, and fluorouracil or epirubicin, cisplatin, and capecitabine group (85% vs 74%; *P* = .02).^36^ In the present trial, the R0 resection rate was 75% (95% CI, 60.4%-86.4%) in the full analysis set, which could be explained by the more advanced stages of disease compared with the previous studies in which approximately 90% of patients were in stage cT4 (eTable 5 in Supplement 1) and a high proportion of patients who did not receive surgery. However, the desired effectiveness was achieved in the per-protocol set with an R0 resection rate of 90% (95% CI, 76.3%-97.2%).

The response rate, including radiologic response and pathological response to treatment, is one of the secondary end points. RECIST 1.1 is one of the most commonly used guidelines for the radiologic assessment of tumor response in advanced GC. According to RECIST 1.1, the radiologic response rate of 75.0% was high, and the proportion of patients with T downstaging was also higher (36.4%). Pathological response is another objective assessment method. Whether histologic regression is a prognostic marker for survival in patients with gastric cancer is still controversial, but cumulative evidence has supported the use of tumor regression as a predictive parameter of survival outcomes.^28,37,38^ In this trial, 26 patients (54.2%) were evaluated as having a pathological response, and 12 patients (25.0%) were evaluated as having a major pathological response. These data confirm the effectiveness of apatinib plus chemotherapy. We also found that the patients with tumors located in the upper one-third of the stomach had a significantly higher pathological response rate, which may be associated with their unique biological characteristics. Furthermore, an Eastern Cooperative Oncology Group Performance Status score of 0 was significantly associated with a higher pathological response rate, suggesting that the performance status may be associated with the treatment response. These results could provide evidence to support the identification of patients in future studies for whom apatinib plus SOX may be more beneficial.

Neoadjuvant chemotherapy seems to have an association with surgical morbidity and mortality. In the European Organisation for Research and Treatment of Cancer 40954 trial, Schuhmacher et al^39^ reported that postoperative complications were more frequent after neoadjuvant chemotherapy compared with surgery alone (27.1% vs 16.2%; *P* = .09). This pattern was also observed in the FNCLCC/FFCD 9703 study.^35^ Thus, the safety of D2 gastrectomy after neoadjuvant treatment for locally advanced GC appeared to be of particular concern for surgeons. In the present study, all of the patients in the 2 treatment groups who underwent gastrectomy underwent standard D2 lymphadenectomy. The incidences of postoperative complications were 18.4%. All complications were evaluated as a Clavien-Dindo grade II, and no postoperative deaths were observed. This result is consistent with that of the study by Shrikhande et al.^40^ D2 gastrectomy after neoadjuvant treatment can be safely performed in patients with locally advanced GC. The patients who achieved pathological response had significantly less blood loss and significantly more lymph node harvested, suggesting that achieving a pathological response was associated with better surgical outcomes.

As previously reported, hypertension, hand-foot syndrome, and proteinuria were the most common AEs of antiangiogenic agents.^19,41,42^ In this study, hypertension occurred in only 16 patients (33.3%), hand-foot syndrome in 10 patients (20.8%), and proteinuria in 9 patients (18.8%). In contrast, the most common AEs of apatinib plus SOX were neutropenia (45.8%), leukopenia (43.8%), increased transaminase (41.7%), and anemia (37.5%). These data showed that the toxic effects of apatinib combined with SOX were mostly SOX-associated toxic effects. Similar to the study by Zhao et al^43^ that used the SOX regimen, most toxic effects were grade 1 or 2, and 33.3% of patients experienced grade 3 toxic effects without chemotherapy-associated death or serious complications. We believe that the findings from the present study support the good tolerability profile of apatinib plus chemotherapy.

No consensus has been reached regarding the optimal number of neoadjuvant treatment cycles for locally advanced GC. In a randomized 2-by-2 factorial clinical trial, patients with resectable advanced GC were assigned to receive 2 or 4 courses of cisplatin with S-1 or docetaxel with cisplatin plus S-1 as neoadjuvant chemotherapy. The R0 resection rates and pathological response rates were comparable between patients who received 2 courses and those who received 4 courses regardless of the regimen used.^44^ In the present trial, most pathological responses and major pathological responses were achieved in patients who completed 3 cycles, which could be partially explained by the fact that patients who achieved a good tumor response were more likely to undergo surgery after completing 3 cycles. These results also suggested that those who were treatment-sensitive would achieve a good tumor response after receiving 3 cycles; however, additional cycles of neoadjuvant treatment were not associated with a higher response rate for those who were treatment-resistant. Three preoperative cycles of apatinib plus SOX will be administered to patients in the future phase 3 of this trial.

### Limitations

This study has some limitations. Selection bias could not be ruled out because of the single-group design. In addition, the sample size was not large. Nevertheless, the final results show the effectiveness and safety of apatinib combined with SOX. A large, multicenter, randomized clinical trial could confirm the advantage of apatinib combined with SOX in neoadjuvant treatment for locally advanced GC.

## Conclusions

Apatinib plus SOX appeared to be a safe, effective, and well-tolerated regimen of neoadjuvant treatment in patients with locally advanced GC. A large-scale randomized clinical trial is needed to confirm this conclusion.

## Notes

Supplement 1. **eTable 1.** Eligibility Criteria for Enrolling Patients **eTable 2.** The Distribution of Multicenter Patients **eTable 3.** Tumor Stage According to Neoadjuvant Chemotherapy Administration **eTable 4.** Comparison of Baseline Characters Between the Patients Achieving and Not Achieving Pathological Response **eTable 5.** Comparison of Baseline Clinical Stages and R0 Resection Rates Between the Present Study and the Previous Studies eReferences **eFigure 1.** Field of Lymph Node Dissection **eFigure 2.** Radiologic Response Assessment **eFigure 3.** Pathological Response Assessment **eFigure 4.** Pathological Response Among Patients Proceeding to Surgery **eFigure 5.** The Pathological Responses in Patients Proceeding to Surgery According to Treatment Cycles **eFigure 6.** Pathological Response According to Baseline Characters in Logistic Regression Model

Supplement 2. Trial Protocol

Supplement 3. Data Sharing Statement
