# Supplementary material for: Effectiveness and Safety of Apatinib Plus Chemotherapy as Neoadjuvant Treatment for Locally Advanced Gastric Cancer: A Nonrandomized Controlled Trial
Source: JAMA Netw Open. 2021 Jul 9;4(7):e2116240. doi: 10.1001/jamanetworkopen.2021.16240 (PMC8271357; doi:10.1001/jamanetworkopen.2021.16240)
Supplement: Supplement 1. — eTable 1. Eligibility Criteria for Enrolling Patients eTable 2. The Distribution of Multicenter Patients eTable 3. Tumor Stage According to Neoadjuvant Chemotherapy Administration eTable 4. Comparison of Baseline Characters Between the Patients Achieving and Not Achieving Pathological Response eTable 5. Comparison of Baseline Clinical Stages and R0 Resection Rates Between the Present Study and the Previous Studies eReferences eFigure 1. Field of Lymph Node Dissection eFigure 2. Radiologic Response Assessment eFigure 3. Pathological Response Assessment eFigure 4. Pathological Response Among Patients Proceeding to Surgery eFigure 5. The Pathological Responses in Patients Proceeding to Surgery According to Treatment Cycles eFigure 6. Pathological Response According to Baseline Characters in Logistic Regression Model [file jamanetwopen-e2116240-s001.pdf]

Supplementary Online Content

Lin JX, Xu YC, Lin W, et al. Effectiveness and safety of apatinib plus chemotherapy as neoadjuvant treatment for locally advanced gastric cancer: a nonrandomized controlled trial. *JAMA Netw Open*. 2021;4(7):e2116240. doi:10.1001/jamanetworkopen.2021.16240

eTable 1. Eligibility Criteria for Enrolling Patients

eTable 2. The Distribution of Multicenter Patients

eTable 3. Tumor Stage According to Neoadjuvant Chemotherapy Administration

eTable 4. Comparison of Baseline Characters Between the Patients Achieving and Not Achieving Pathological Response

eTable 5. Comparison of Baseline Clinical Stages and R0 Resection Rates Between the Present Study and the Previous Studies

eReferences

eFigure 1. Field of Lymph Node Dissection

eFigure 2. Radiologic Response Assessment

eFigure 3. Pathological Response Assessment

eFigure 4. Pathological Response Among Patients Proceeding to Surgery

eFigure 5. The Pathological Responses in Patients Proceeding to Surgery According to Treatment Cycles

eFigure 6. Pathological Response According to Baseline Characters in Logistic Regression Model

This supplementary material has been provided by the authors to give readers additional information about their work.

eTable 1. Eligibility Criteria for Enrolling Patients

Inclusion
Age from 18 to 75 years
Primary gastric adenocarcinoma (papillary, tubular, mucinous, signet ring cell, or poorly differentiated) confirmed pathologically by endoscopic biopsy
cT2-4/N+M0 at preoperative evaluation according to the American Joint Committee on Cancer (AJCC) Cancer Staging Manual Seventh Edition
No distant metastasis is observed. And the spleen, pancreas or other adjacent organs are not involved by the tumor
Performance status of 0 to 2 on Eastern Cooperative Oncology Group scale
Without previous surgery, chemotherapy, radiotherapy, or targeted therapy
Estimate life is equal or more than 3 months
No serious heart, lung or liver dysfunction; no jaundice or obstruction of the digestive tract; no acute infection
The main organ function is normal, and meet the following criteria:
blood routine examination (No blood transfusion within 14 days)
Hb ≥ 100g/L,<br>WBC ≥ 3.5×10^9/L,<br>ANC ≥ 1.5×10^9/L,<br>PLT ≥ 100×10^9/L;
blood biochemical examination
BIL<1.5 ULN,<br>ALT and AST ≤ 2.5×ULN, GGT ≤ 1.5×ULN,<br>Cr ≤ 1×ULN, creatinine clearance ＞60ml/min (Cockcroft-Gault formula)
Written informed consent
Exclusion
Pregnant and lactating women
Severe mental disorder

© 2021 Lin JX et al. *JAMA Network Open.*

History of previous upper abdominal surgery (except for laparoscopic cholecystectomy)
History of previous chemotherapy or radiotherapy
History of other malignant disease within the past 5 years
History of unstable angina or myocardial infarction within the past 6 months
History of cerebrovascular accident within the past 6 months
History of continuous systematic administration of corticosteroids within 1 month
Emergency surgery due to complication (bleeding, obstruction or perforation)
Patients with a clear tendency of gastrointestinal bleeding, such as: active ulceration, fecal occult blood test(++)
History of hematemesis and melena within 2 months, coagulation disorders
Positive urinary protein (uric albumin (++),or 24-hour urinary protein > 1.0g)
Factors affecting oral administration, such as dysphagia, uncontrollable nausea and vomiting, chronic diarrhea, and intestinal obstruction
Drug allergy to experimental medicine
Abbreviation: Hb = Hemoglobin, WBC = White blood cell, ANC = Absolute neutrophil count, PLT = Platelet, BIL = Bilirubin, ALT = Alanine aminotransferase,
AST = Aspartate aminotransferase, GGT = Glutamyl transferase, Cr = Creatinine,
INR = International Normalized Ratio, APTT = Activated partial thromboplastin time
ULN = Upper limit of normal.

© 2021 Lin JX et al. *JAMA Network Open.*

eTable 2. The Distribution of Multicenter Patients

Center	Cases
Fujian Medical University Union Hospital	16
The First Hospital of Putian	10
The Affliated Hospital of Putian University	6
Fujian Provincial Hospital	4
The First Affiliated Hospital of Fujian Medical University	3
Fujian Provincial Cancer Hospital	3
Zhangzhou Affiliated Hospital of Fujian Medical University	2
The First Affiliated Hospital of Xiamen University	2
The Second Affiliated Hospital of Fujian Medical University	1
Zhongshan Hospital Affiliated to Xiamen University	1

© 2021 Lin JX et al. *JAMA Network Open.*

eTable 3. Tumor Stage According to Neoadjuvant Chemotherapy Administration

	**Pre-chemotherapy**	**Post-chemotherapy**
**Apatinib+SOX(n=44)**		
cT stage		
T0	0(0.0)	0(0.0)
T1	0(0.0)	2(4.5)
T2	0(0.0)	4(9.1)
T3	4(9.1)	14(31.8)
T4	40(90.9)	24(54.5)
Change in cT stage		
Downstaged	16(36.4)	
No change/Upstaged	28(63.6)	
cN stage		
N0	3(6.8)	7(15.9)
N+	41(93.2)	37(84.1)
Change in cN stage		
Downstaged	4(9.8)	
No change/Upstaged	37(90.2)	

© 2021 Lin JX et al. *JAMA Network Open.*

eTable 4. Comparison of Baseline Characters Between the Patients Achieving and Not Achieving Pathological Response

**Variable**	**Tumor regression < 1/3 (n=22)**	**Tumor regression ≥ 1/3 (n=26)**	**P value**
Age, years			0.317
Median	67	63	
Range	41-75	41-74	
Sex			0.473
Male	18(81.8)	19(73.1)	
Female	4(18.2)	7(26.9)	
Body Weight Index			0.331
Median	21	22	
Range	17.2-27.9	15.1-30.9	
ECOG PS			**0.025**
0	10(45.5)	20(76.9)	
1/2	12(54.5)	6(23.1)	
Tumor differentiation			0.936
Well/moderately differentiated	5(22.7)	7(26.9)	
Poorly differentiated/mucinous or signet ring cell carcinoma	12(54.5)	13(50.0)	
Unknown	5(22.7)	6(23.1)	
Lauren classification			
Intestinal	5(22.7)	6(23.1)	
Diffuse	12(54.5)	14(53.8)	
Unknown	5(22.7)	6(23.1)	
Tumor location			**0.040**
Upper 1/3	7(31.8)	16(61.5)	
Middle or Lower 1/3	15(68.2)	10(38.5)	
Borrmann type			-
I/II	1(4.5)	2(7.7)	
III	19(86.4)	23(88.5)	
IV	2(9.1)	1(3.8)	
cT stage			0.419
T3	3(13.6)	2(7.7)	
T4	19(86.4)	24(92.3)	
cN stage			0.371
N0	1(4.5)	3(11.5)	
N+	21(95.5)	23(88.5)	
Abbreviation: ECOG=Eastern Cooperative Oncology Group.			

© 2021 Lin JX et al. *JAMA Network Open.*

eTable 5. Comparison of Baseline Clinical Stages and R0 Resection Rates Between the Present Study and the Previous Studies

Trials	Regimen	cT stage	cN stage	R0 resection rate
Al-Batran SE, et al. [1]	ECF/ECX	cT1-2: 18%	cN0: 20%	74%
		cT3-4: 82%	cN+: 80%	
	FLOT4	cT1-2: 18%	cN0: 23%	85%
		cT3-4: 81%	cN+: 77%	
Wang X, et al. [2]	mFOLFOX6	cT2: 12.3%	cN0: 30.1%	91.8%
		cT3: 57.5%	cN+: 69.9%	
		cT4: 20.1%		
Oki E, et al. [3]	DS	cT2: 8.5%	cN0: 2.1%	93.6%
		cT3: 80.9%	cN1: 63.8%	
		cT4: 10.6%	cN2: 34.1%	
**Present**	**Apatinib+SOX**	**cT3: 10.4%**	**cN0: 8.3%**	**75%**
		**cT4: 89.6%**	**cN+: 91.7%**	

© 2021 Lin JX et al. *JAMA Network Open.*

eReferences

1. Al-Batran SE, Hofheinz RD, Pauligk C, et al. Histopathological regression after neoadjuvant docetaxel, oxaliplatin, fluorouracil, and leucovorin versus epirubicin, cisplatin, and fluorouracil or capecitabine in patients with resectable gastric or gastro-oesophageal junction adenocarcinoma (FLOT4-AIO): results from the phase 2 part of a multicentre, open-label, randomised phase 2/3 trial. Lancet Oncol. 2016; 17: 1697-1708.

2. Wang X, Zhao L, Liu HF, et al. A phase II study of a modified FOLFOX6 regimen as neoadjuvant chemotherapy for locally advanced gastric cancer. Br. J. Cancer. 2016; 114: 1326-1333.

3. Oki E, Emi Y, Kusumoto T, et al. Phase II study of docetaxel and S-1 (DS) as neoadjuvant chemotherapy for clinical stage III resectable gastric cancer. Ann. Surg. Oncol. 2014; 21: 2340-2346.

© 2021 Lin JX et al. *JAMA Network Open.*

eFigure 1. Field of Lymph Node Dissection

(a) Inferior pylorus region; (b) Superior margin of the pancreas; (c) Splenic hilus region.

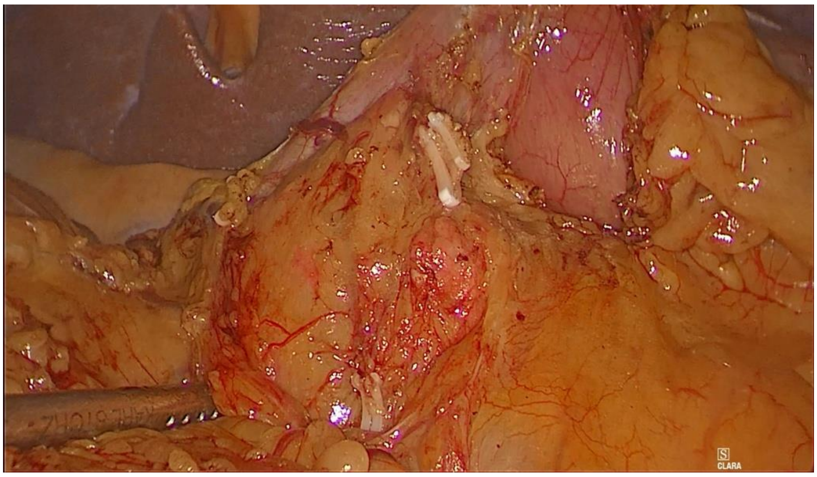

(a)

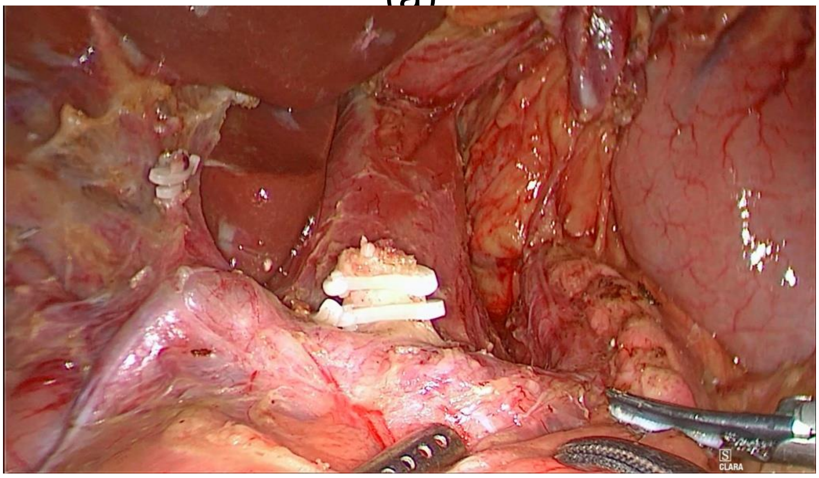

(b)

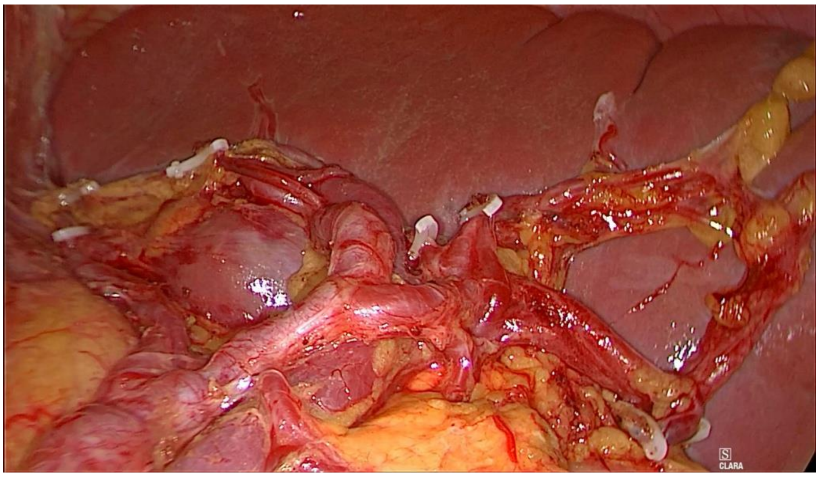

(c)

© 2021 Lin JX et al. *JAMA Network Open.*

eFigure 2. Radiologic Response Assessment

White Arrows Illustrate a Pathological Node With the Short Axis Shown as a Dotted Line at Baseline and at the Last Follow-up Before Surgery in the Following Images. (a) The length of the short axis is 35.1mm in the left image, and 15.1mm in the right; this case was considered as partial response (PR). (b) The length of the short axis is 21.1mm in the left image, and 12.3mm in the right; this case was considered as PR. (c) The length of the short axis is 17.0mm in the left image, and the node was not found in the right; this case was considered as complete response (CR).

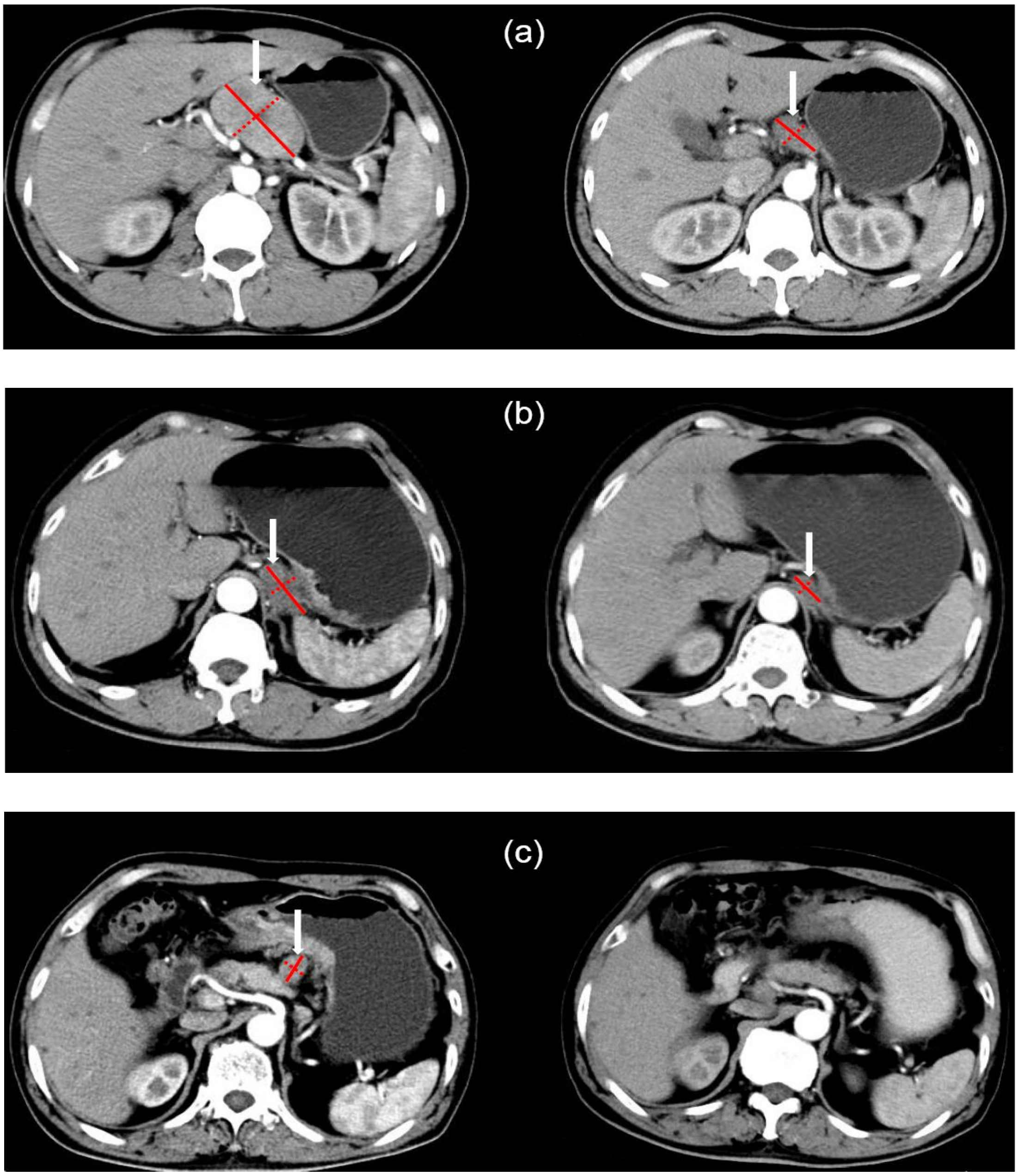

© 2021 Lin JX et al. *JAMA Network Open.*

eFigure 3. Pathological Response Assessment

(a) Grade 1a, the degeneration area was less than 1/3; (b) grade 1b, the degeneration area was 1/3–2/3; (c) grade 2a, the degeneration area was 2/3–9/10; (d) grade 2b, the degeneration area was more than 9/10; and (e) grade 3, no residual tumor.

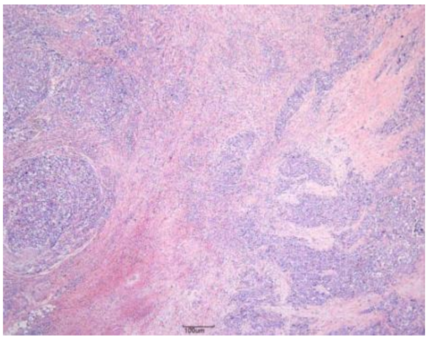

(a)

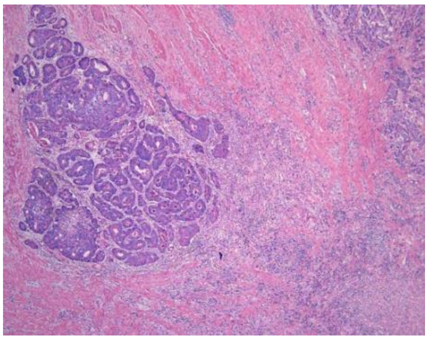

(b)

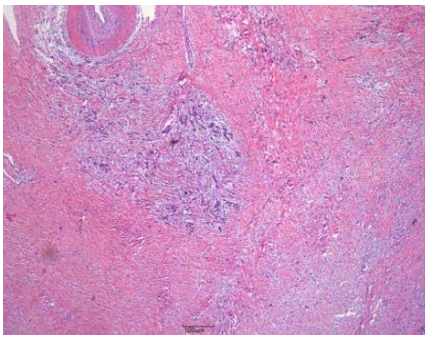

(c)

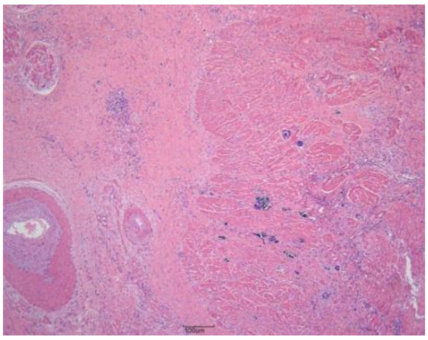

(d)

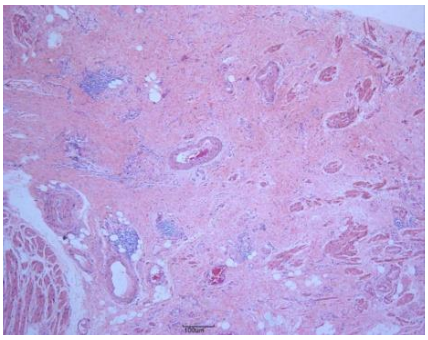

(e)

© 2021 Lin JX et al. *JAMA Network Open.*

eFigure 4. Pathological Response Among Patients Proceeding to Surgery

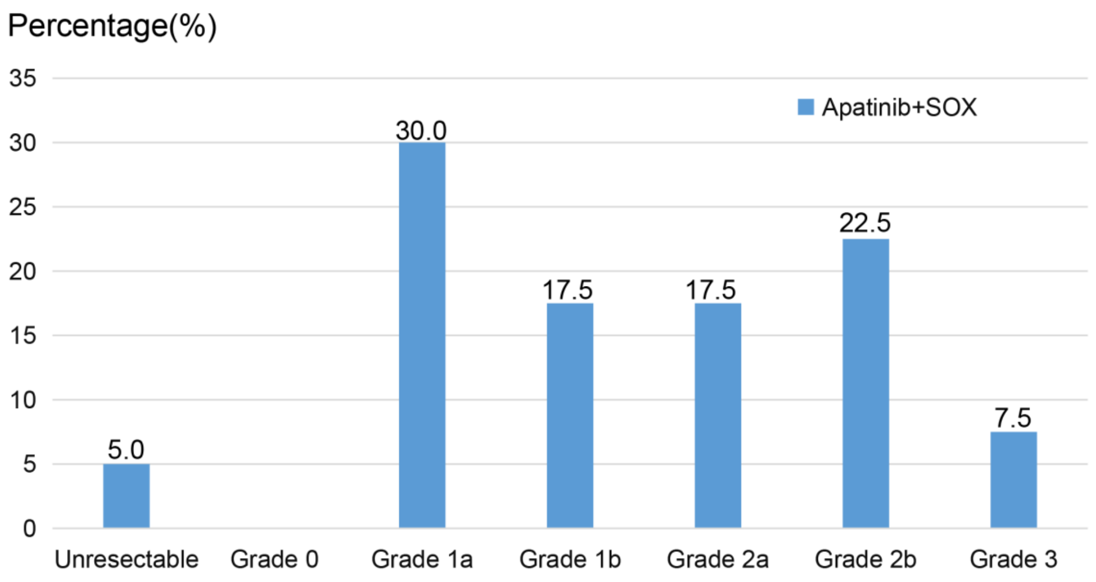

© 2021 Lin JX et al. *JAMA Network Open.*

eFigure 5. The Pathological Responses in Patients Proceeding to Surgery According to Treatment Cycles

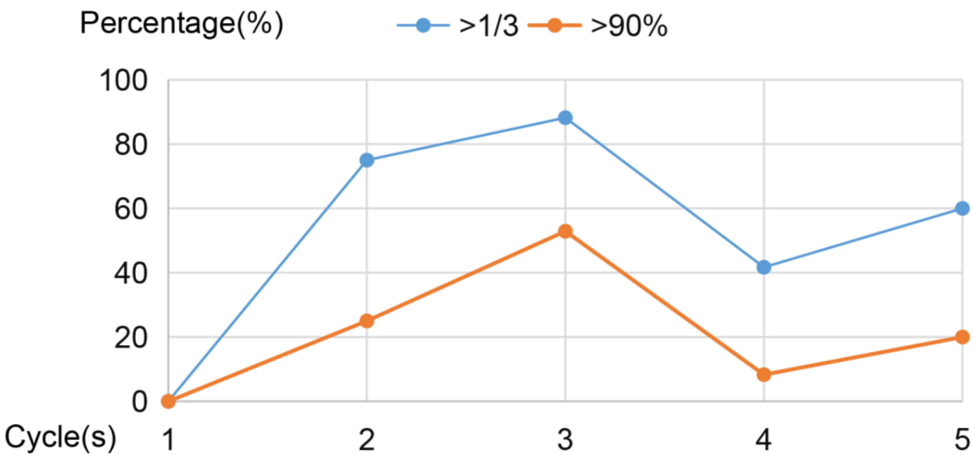

Tumor regression > 1/3	0/2	3/4	15/17	5/12	3/5
Tumor regression > 90%	0/2	1/4	9/17	1/12	1/5

© 2021 Lin JX et al. *JAMA Network Open.*

eFigure 6. Pathological Response According to Baseline Characters in Logistic Regression Model

Variables	Hazard ratio(95% CI)	P value
Age		
Per 10 years	0.692(0.338-1.416)	0.313
Gender		
Male *vs* Female	1.658(0.414-6.639)	0.475
BMI		
Per 1 kg/m2	1.089(0.919-1.290)	0.325
ECOG PS		
1/2 *vs* 0	0.250(0.072-0.864)	**0.028**
Tumor differentiation		
Poorly differentiated *vs* Well/moderately differentiated	0.774(0.193-3.107)	0.718
Tumor differentiation		
Unknown *vs* Well/moderately differentiated	0.857(0.164-4.467)	0.855
Lauren classification		
Diffuse *vs* Intestinal	0.972(0.236-4.003)	0.969
Lauren classification		
Unknown *vs* Intestinal	1.000(0.187-5.357)	1.000
Tumor location		
Middle or Lower 1/3 *vs* Upper 1/3	0.292(0.088-0.964)	**0.043**

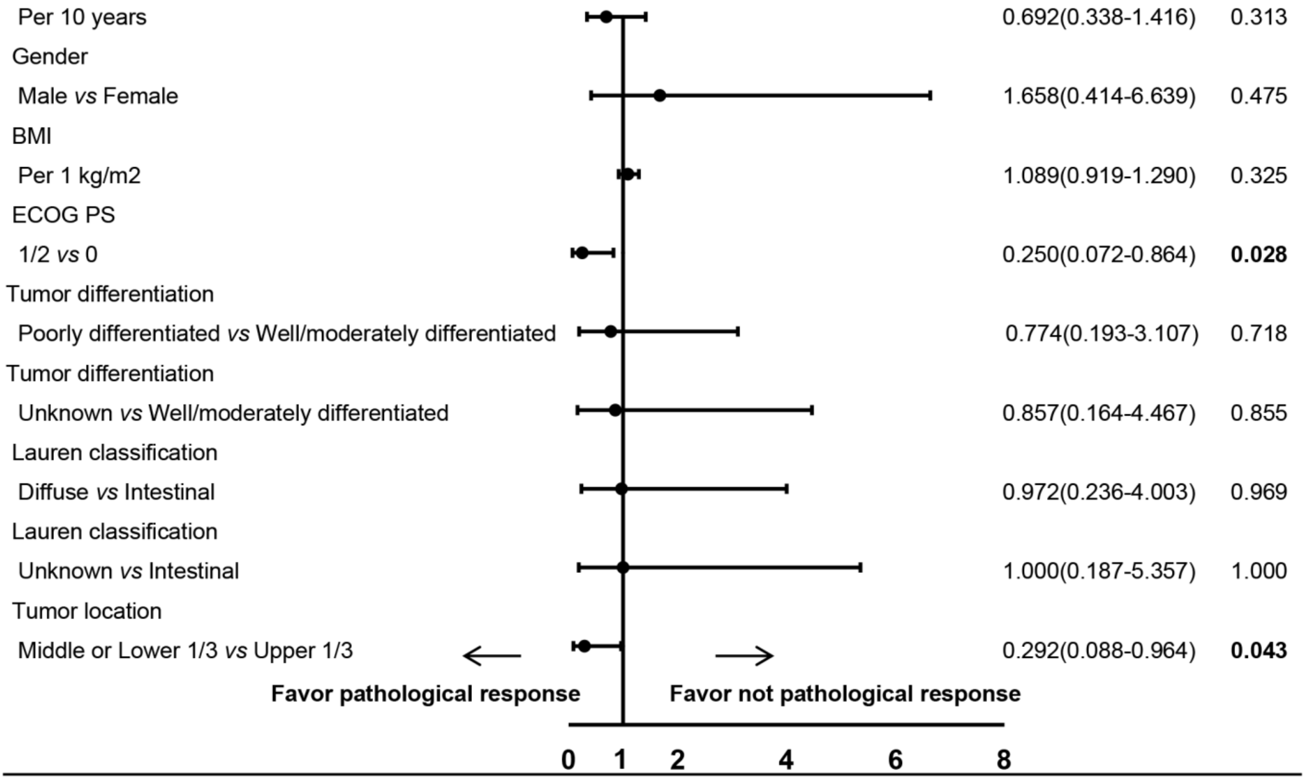

Abbreviation: BMI = Body mass index. ECOG = Eastern Cooperative Oncology Group. PS = Performance status.

© 2021 Lin JX et al. *JAMA Network Open.*
