# Supplementary material for: Effectiveness and Safety of Apatinib Plus Chemotherapy as Neoadjuvant Treatment for Locally Advanced Gastric Cancer: A Nonrandomized Controlled Trial
Source: JAMA Netw Open. 2021 Jul 9;4(7):e2116240. doi: 10.1001/jamanetworkopen.2021.16240 (PMC8271357; doi:10.1001/jamanetworkopen.2021.16240)
Supplement: Supplement 2. — Trial Protocol [file jamanetwopen-e2116240-s002.pdf]

Efficacy and safety of apatinib combined with S-1 plus oxaliplatin as neoadjuvant treatment for locally advanced gastric cancer: A multicenter, prospective study (Arise-FJ-G001)

Study protocol

Bidding party: Fujian Medical University Union Hospital

Principle Investigator:

Prof. Chang-Ming Huang, M.D. Ph.D.

Department of Gastric Surgery, Fujian Medical University Union Hospital,

Address: No. 29 Xinquan Road, Fuzhou 350001 Fujian Province, China.

Telephone: +86-591-83363366, Fax: +86-591-83363366

No. of edition: V2.0

The date of the edition: 2017.04.06

Summary

Scenario Title	Efficacy and safety of apatinib combined with S-1 plus oxaliplatin as neoadjuvant treatment for locally advanced gastric cancer: A multicenter, prospective study (Arise-FJ-G001)
Scenario Version	V2.0
Principle Investigator	Chang-Ming Huang
Bidding Center	Fujian Medical University Union Hospital
Study Population	Patients with locally advanced gastric cancer (cT2-4/N+M0)
Purpose of Research	To investigate the efficacy and safety of apatinib combined with S-1 plus oxaliplatin (SOX) as a neoadjuvant treatment for locally advanced gastric cancer.
Research Design	Multicenter, single-arm, prospective
Case Grouping	Single group
Sample Size	50 cases
Inclusion Criteria	● Age from 18 to 75 years<br>● Primary gastric adenocarcinoma (papillary, tubular, mucinous, signet ring cell, or poorly differentiated) confirmed pathologically by endoscopic biopsy<br>● cT2-4/N+M0 at preoperative evaluation according to the American Joint Committee on Cancer (AJCC) Cancer Staging Manual Seventh Edition<br>● No distant metastasis is observed. And the spleen, pancreas or other adjacent organs are not involved by the tumor.<br>● Performance status of 0 to 2 on Eastern Cooperative Oncology Group scale (ECOG)

	● Without previous surgery, chemotherapy, radiotherapy, immunotherapy, or targeted therapy for gastric cancer.<br>● Estimate life is equal or more than 3 months<br>● No serious heart, lung, liver dysfunction; no jaundice or obstruction of the digestive tract; no acute infection<br>● The main organ function is normal, and meet the following criteria:<br>blood routine examination(No blood transfusion within 14 days)<br>HB≥100g/L，<br>WBC≥3.5×109/L,<br>ANC≥1.5×109/L，<br>PLT≥100×109/L；<br>blood biochemical examination<br>BIL<1.5 ULN，<br>ALT and AST<2.5ULN，GGT≤1.5×ULT；<br>Cr≤1ULN，creatinine clearance＞60ml/min （Cockcroft-Gault formula）<br>● Written informed consent
Exclusion Criteria	● Pregnant and lactating women<br>● Severe mental disorder<br>● History of previous upper abdominal surgery (except for laparoscopic cholecystectomy)<br>● History of previous chemotherapy or radiotherapy<br>● History of other malignant disease within the past 5 years<br>● History of unstable angina or myocardial infarction within the past 6 months<br>● History of cerebrovascular accident within the past 6 months<br>● History of continuous systematic administration of corticosteroids within 1 month

	● Emergency surgery due to complication (bleeding, obstruction or perforation) caused by gastric cancer<br>● Patients with a clear tendency of gastrointestinal bleeding, such as: active ulceration,fecal occult blood test(++). History of hematemesis and melena within 2 months, coagulation disorders (INR>1.5、APTT>1.5 ULN).<br>● Positive urinary protein (uric albumen check(++), or 24-hour urinary protein content>1.0g)<br>● Factors affecting oral administration, such as dysphagia, uncontrollable nausea and vomiting, chronic diarrhea, and intestinal obstruction<br>● Drug allergy to experimental medicine
Rejection Criteria	● Do not meet the inclusion criteria and meet the exclusion criteria;<br>● Simultaneous application of CFDA-approved modern Chinese medicine or immunomodulators for the treatment of gastric cancer;<br>● Radiation therapy or other local treatments during the study periods;<br>● Incomplete data;<br>● Do not take medicine according to the dosage, method, and treatment prescribed in the protocol.
Criteria for Stopping Treatment	● Receiving surgery;<br>● Disease progression;<br>● Unbearable toxicity after dose reductions;<br>● Patient refusal;<br>● Investigator decision that stopping treatment was in the best interest of the patient;<br>● Death.
Intervention	● Oxaliplatin, 130 mg/m2 intravenously on day 1;<br>● Apatinib, 500 mg orally once daily on days 1 to 21;<br>● S-1 (Tigio), 40-60 mg orally twice daily on days 1 to 14.<br>The dose of S-1 is based on body surface area (BSE):

<table>
<tr>
<td></td>
<td>
<table>
<tr><th>BSE</th><th>Dose</th></tr>
<tr><td>< 1.25m^2</td><td>40mg× 2/day</td></tr>
<tr><td>1.25m^2 - 1.5m^2</td><td>50mg× 2/day</td></tr>
<tr><td>> 1.5m^2</td><td>60mg× 2/day</td></tr>
</table>
●
</td>
</tr>
<tr>
<td>Outcome Measures</td>
<td>
Primary Outcome Measures:
● R0 resection rate
Secondary Outcome Measures:
● Pathological response rate
● Radiologic response rate
● Surgical safety: including surgical mortality, surgical morbidity (bleeding, anastomotic leakage, wound infection), reoperation rate, length of stay, etc.
● Drug safety: including adverse events (AEs), severe adverse events (SAEs), and drug-related AEs (like hypertension, hand-foot syndrome, and proteinuria).
● Disease-free survival (DFS)
● Overall survival (OS)
</td>
</tr>
</table>

Flow Chart

Items	Screening Period		Neoadjuvant Treatment Period									Surgery		Adjuvant Treatment Period		
	Within 2 weeks before enrollment	Within 1 weeks before enrollment	Cycle 1	Cycle 2		Cycle 3		Cycle 4		Cycle 5		Within 1 weeks before surgery	Within 1 weeks after surgery	Cycle 1		Cycles 2-6
			Day 7 (±3)	Day 1 (±3)	Day 7 (±3)	Day 1 (±3)	Day 7 (±3)]	Day 1 (±3)	Day 7 (±3)	Day 1 (±3)	Day 7 (±3)			Day 1 (±3)	Day 7 (±3)	Day 1 (±3)
**Baseline characters**																
Informed consent	×															
Demographics characters	×															
History of other diseases	×															
Vital signs		×		×		×		×		×		×	×	×		×
Physical examination		×		×		×		×		×		×	×	×		×
**Laboratory tests**																
Peripheral blood routine		×	×	×	×	×	×	×	×	×	×	×	×	×	×	×
Urinary routine		×		×		×		×		×		×		×		×
Stool routine		×		×		×		×		×		×		×		×
Blood chemistry		×	×	×	×	×	×	×	×	×	×	×	×	×	×	×
Coagulation tests		×		×		×		×		×		×		×		×
Tumor markers		×		×		×		×		×		×		×		×

**Tumor sample collection**													×			
**Gastroscopy**		×														
**Electrocardiograms**		×		×		×		×		×		×		×		×
**Pregnancy tests**		×														
**Radiologic assessments**																
**Imaging examination]**	×		**Every 2 cycles (±7)**													×
**Other assessments**																
**ECOG score**		×		×		×		×		×		×	×	×		×
**Blood pressure monitoring**		×	×	×	×	×	×	×	×	×	×	×	×	×	×	×
**Adverse events**			×	×	×	×	×	×	×	×	×	×	×	×	×	×
**Others**																
**Concomitant drugs**			×	×	×	×	×	×	×	×	×	×	×	×	×	×
**Drug compliance**				×		×		×		×				×		×

22

23 Abbreviations

Abbreviations	Full names
AE	Adverse events
AGC	Advanced gastric cancer
AKP	Alkaline phosphatase
ALB	Albumin
ALT	Alanine aminotransferase
ANC	Absolute neutrophil count
APTT	Activated partial thromboplastin time
AST	Aspartate aminotransferase
Bid	Bis in die
BIL	Bilirubin
BSE	Body surface area
BUN	Blood urea nitrogen
CFDA	China Food and Drug Administration
CI	Confidence interval
Cr	Creatinine
CR	Complete response
CRF	Case Report Form
CT	Computed tomography
DFS	Disease-free survival
DRQ	Data Request Question
ECOG PS score	Eastern Cooperative Oncology Group scale Performance status score
FAS	Full Analysis Set
Fbg	Fibrinogen
GCP	Good Clinical Practice

Abbreviations	Full names
Hb	Hemoglobin
ICF	Informed consent form
INR	International Normalized Ratio
IRB	Institutional Review Board
LDH	Lactate dehydrogenase
MRI	Magnetic resonance imagin
NCI-CTC	National Cancer Institute - Common Terminology Criteria
OS	Overall survival
PET	Positron emission tomography
PD	Progressive Disease
PLT	Platelet
PR	Partial Response
PT	Prothrombin time
Qd	Quaque die
Qod	Quaque secundo die
QoL	Quality of Life
RBC	Red blood cells
RECIST	Response Evaluation Criteria in Solid Tumors
SAE	Severe adverse events
SD	Stable Disease
SOC	System organ classification
TBIL	Total bilirubin
TP	Total protein
TT	Thrombin time
ULN	Upper limit of normal
VEGFR	Vascular endothelial growth factor receptor
WBC	White blood cells
γ-GT	Glutamyl transferase

1. Background

There were over 1,000,000 new cases and an estimated 783,000 deaths caused by gastric cancer in 2018, making it the fifth most frequently diagnosed cancer and the third leading cause of cancer death [1]. Endoscopic or surgical resection is curative in most early gastric cancers, with a 5-year overall survival (OS) rate greater than 90% [2]. In contrast, prognosis remains poor for locally AGC even after the complete dissection of the primary tumors and regional lymph nodes [3]. Thus, multimodal treatments have been proposed to prolong the survival period. Since the MAGIC trial published in 2006 first demonstrated that perioperative chemotherapy (ECF) could increase the 5-year OS rate from 23% to 36% compared with surgery alone ($P = 0.009$) [4], systemic chemotherapy has become the standard treatment for locally AGC [5]. In Asia, oral fluoropyrimidines (e.g., S-1 or capecitabine) plus oxaliplatin is considered a first-line regimen [6-7]. However, chemotherapy was reported to be less effective against gastric cancer than against other solid malignancies because of the heterogeneity of tumors [8-9]. Therefore, new treatment options showing promising efficacy and acceptable safety profiles are urgently needed.

In the past decade, several clinical trials have been performed to investigate molecular targeted therapy for AGC, but few molecular agents have shown promising activity [10-13]. The ToGA trial demonstrated that trastuzumab with cisplatin and capecitabine or 5-FU (XP/FP) was associated with improved OS for HER2-positive advanced G/GEJ cancer [10]. Unfortunately, only a small percentage of patients (approximately 20%) are ideal candidates for HER-2 targeted therapy [10, 14-15]. Another well-established target is vascular endothelial growth factor (VEGF). VEGF is one of the most potent angiogenic factors and is a signaling molecule secreted by many solid tumors [16-17]. Since high VEGF expression is one of the characteristic features of gastric carcinomas, targeting VEGF is therefore considered a promising therapeutic strategy.

Apatinib, a novel receptor tyrosine kinase inhibitor selectively targeting VEGFR-2, strongly inhibited VEGF-mediated endothelial cell migration, proliferation, and tumor microvascular density [18]. A phase III study showed that apatinib improved OS and progression-free survival (PFS) in patients with chemotherapy-refractory advanced or metastatic adenocarcinoma of the stomach or gastroesophageal junction (GEJ) when compared with placebo [19].

Neoadjuvant chemotherapy could decrease the tumor stage, increase the R0 resection rate and the survival benefits for AGC patients [20-21]. However, rare evidence supports the utility of apatinib combined with chemotherapy in neoadjuvant treatment. Thus, a multicenter, prospective study was conducted to investigate the efficacy and safety of apatinib plus SOX for the neoadjuvant treatment of locally AGC.

2. Objective

2.1 Primary End Point

R0 resection rate

2.2 Secondary End Point

Pathological / radiologic response rate, toxicity, and surgical outcome

3. Research Pattern and Design

3.1 Research Pattern

This is a multicenter, single-arm, prospective, Phase 2 study.

3.2 Criteria for Study Population

3.2.1 Inclusion criteria

(1) Age from 18 to 75 years

(2) Primary gastric adenocarcinoma (papillary, tubular, mucinous, signet ring cell, or poorly differentiated) confirmed pathologically by endoscopic biopsy

(3) cT2-4/N+M0 at preoperative evaluation according to the American Joint Committee on Cancer (AJCC) Cancer Staging Manual Seventh Edition

(4) No distant metastasis is observed. And the spleen, pancreas or other adjacent organs are not involved by the tumor.

(5) Performance status of 0 to 2 on Eastern Cooperative Oncology Group scale (ECOG)

(6) Without previous surgical, chemotherapy,radiotherapy, immunotherapy, or targeted therapy for gastric cancer.

(7) Estimate life is equal or more than 3 months

(8) No serious heart, lung, liver dysfunction; no jaundice or obstruction of the digestive tract; no acute infection

(9) The main organ function is normal,and meet the following criteria:

blood routine examination (No blood transfusion within 14 days)

HB≥100g/L，

WBC≥3.5×109/L,

ANC≥1.5×109/L，

PLT≥100×109/L；

blood biochemical examination

BIL<1.5 Upper Limit Of Normal（ULN），

ALT and AST<2.5ULN，GGT≤1.5×ULT；

Cr≤1ULN，creatinine clearance＞60ml/min（Cockcroft-Gault formula）

(10) Written informed consent

3.2.2 Exclusion criteria

(1) Pregnant and lactating women

(2) Severe mental disorder

(3) History of previous upper abdominal surgery (except for laparoscopic cholecystectomy)

(4) History of previous chemotherapy or radiotherapy

(5) History of other malignant disease within the past 5 years

(6) History of unstable angina or myocardial infarction within the past 6 months

(7) History of cerebrovascular accident within the past 6 months

(8) History of continuous systematic administration of corticosteroids within 1 month

(9) Emergency surgery due to complication (bleeding, obstruction or perforation) caused by gastric cancer

(10) Patients with a clear tendency of gastrointestinal bleeding,such as: active ulceration, fecal occult blood test(++). History of hematemesis and melena

within 2 months,coagulation disorders (INR>1.5、APTT>1.5 ULN).

(11) Positive urinary protein(uric albumen check(++),or 24-hour urinary protein content>1.0g)

(12) Factors affecting oral administration, such as dysphagia, uncontrollable nausea and vomiting, chronic diarrhea, and intestinal obstruction

(13) Drug allergy to experimental medicine

3.2.3 Rejection criteria

(1) Do not meet the inclusion criteria and meets the exclusion criteria;

(2) Simultaneous application of CFDA-approved modern Chinese medicine or immunomodulators for the treatment of gastric cancer;

(3) Radiation therapy or other local treatments during the study periods;

(4) Incomplete data;

(5) Do not take medicine according to the dosage, method, and treatment prescribed in the protocol.

3.2.4 Criteria for Stopping Treatment

(1) Disease progression;

(2) Unbearable toxicity after dose reductions;

(3) Patient refusal;

(4) Investigator decision that stopping treatment was in the best interest of the patient;

(5) Death.

3.3 Research Design

3.3.1 Technical Route

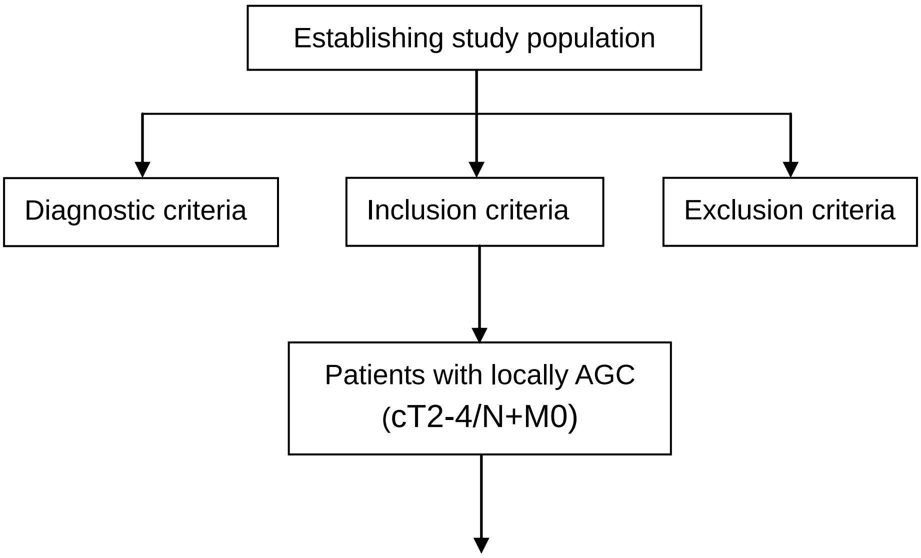

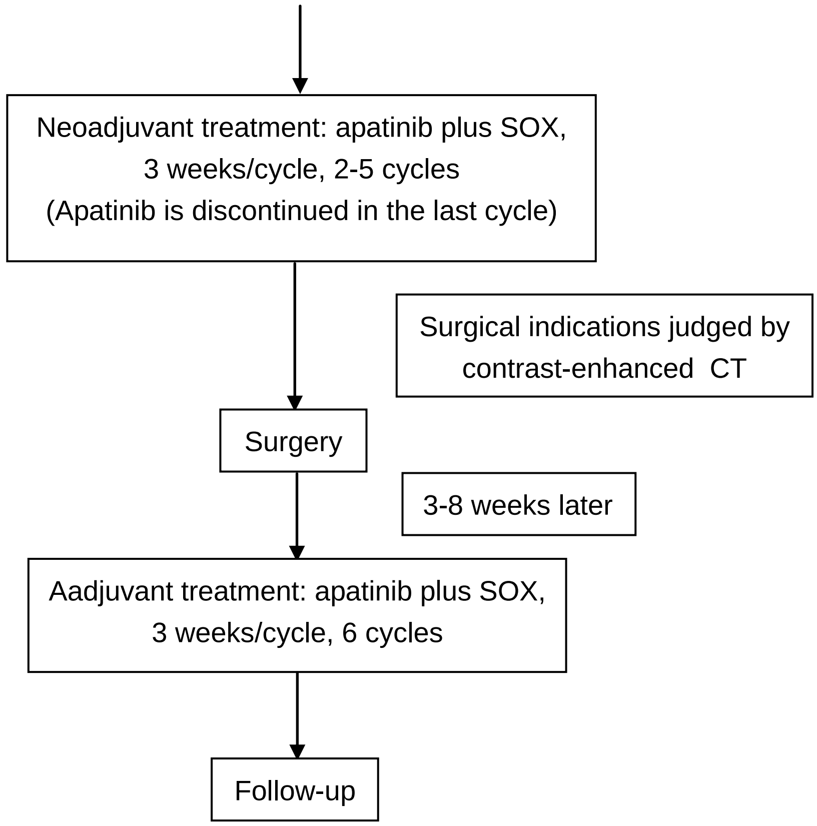

3.3.2 Drug Administration

- Oxaliplatin, 130 mg/m2 intravenously on day 1;
- Apatinib, 500 mg orally once daily on days 1 to 21;
- S-1 (Tigio), 40-60 mg orally twice daily on days 1 to 14.

The dose of S-1 is based on body surface area (BSE):

BSE	Dose
< $1.25m^2$	40mg Bid
$1.25m^2$ - $1.5m^2$	50mg Bid
> $1.5m^2$	60mg Bid

3.4 Research Flow

Start Date: The date on which the written informed consent is signed

End Date: The last day of the post-treatment period (eg, 30 days after the last drug administration).

The expected treatment duration of this study is 36 months, including 24-month enrollment period. Each participants will be followed up from the Start Date to the End Date, and then for at least 5 years.

If some participant receives a new anti-cancer therapy, monitoring of AEs will be terminated at the beginning of this therapy.

3.5 Assessment of Efficacy and Safety

3.5.1 Efficacy Assessment

- Primary Outcome Measures

R0 resection rate

- Secondary Outcome Measures

Pathological response rate: major pathological response rate (G2b+G3, see Appendix 1);

Radiologoc response rate: complete response (CR) + partial response (PR), according to RECIST 1.1;

3.5.2 Safety Assessment

(1) Surgical safety: including surgical mortality, surgical morbidity (bleeding, anastomotic leakage, wound infection), reoperation rate, length of stay, etc.

(2) Drug safety: including AEs, SAEs, and drug-related AEs (e.g. hypertension, hand-foot syndrome, and proteinuria).

3.6 Distribution, Storage and Management of Drugs

Drug distribution must be strict. Special persons from each centers are responsible for distributing and keeping drugs. Researchers should fill in the Registration Forms for Drugs at each visit.

According to the requirements of GCP, study drugs should be uniformly stored, distributed and recycled by research centers. The drugs should be stored at a sealed, protected from light, and room temperature environment. The validity period is tentatively set for 2 years.

4. Study Procedure

4.1 Screening Period

The following screening steps should be completed within 2 weeks before starting treatment:

- Demographics characters: gender, date of birth, ethnicity, height, weight, etc.
- Diagnosis of tumors: date of diagnosis, histological classification, clinical stage, etc.
- History of comorbidity: such as history of diabetes, hypertension or chronic

obstructive pulmonary disease.

- Imaging examination: including whole abdominal computed tomography (CT) or magnetic resonance imaging (MRI), and chest X-ray. Other means of evaluation: gastrointestinal radiography, ultrasonography of other organs, and positron emission tomography (PET)-CT.

After admitting, patients meeting the selection criteria should be screened out. For these patients, researchers will introduce this trial in detail, and the ICF will be signed if the patients agree to participate.

The following screening steps should be completed within 1 weeks before starting treatment:

- ECOG PS score;
- Vital signs: heart rate, breathing rate, temperature, and blood pressure;
- Physical examination: Head and face, skin system, lymph nodes, eyes, ear, nose, throat, mouth, respiratory system, cardiovascular system, abdomen, genitourinary system, musculoskeletal, nervous system, and mental status.
- Peripheral blood routine: Hb, RBC, WBC, ANC, and PLT.
- Urinary routine: Urine protein and occult blood tests; if the semi-quantitative method shows protein ≥2+ (eg, urine test strips), a 24-hour urinary protein quantification test should be performed. The 24-hour urinary protein quantification of enrolled patients must be <1g.
- Stool routine: Occult blood tests;
- Blood chemistry: Total bilirubin, ALT, AST, AKP, r-GT, TP, ALB, BUN, and CA.
- Coagulation tests : PT, APTT, TT, and Fbg;
- Electrocardiograms: 12-lead electrocardiograms;
- Pregnancy tests (for women at childbearing age).

4.2 Neoadjuvant Treatment Period

Intervention group: All enrolled patients receive 2-5 preoperative cycles of apatinib (500 mg orally once daily on days 1 to 21 and discontinued in the last cycle) plus SOX (S-1, 40-60 mg orally twice daily on days 1 to 14; oxaliplatin, 130 mg/m2 intravenously on day 1) every 3 weeks.

- During neoadjuvant treatment, physical examination, ECOG PS assessment, tumor markers, and electrocardiograms are performed on day

1 of cycles 2, 3, 4, and 5. If cardiac pains or palpitations occur, myocardial enzyme spectrum (creatine kinase, lactate dehydrogenase), electrocardiogram, and cardiac ultrasound should be immediately performed;

- Peripheral blood routine and blood chemistry are performed on day 7 of cycle 1 and days 1, 7 of cycles 2, 3, 4, and 5;
- Urinary routine, stool routine, and coagulation tests are performed on day 1 of cycles 2, 3, 4, and 5;
- Imaging examination should be performed under the same conditions (layer thickness of scanning, use of contrast agent, etc.) as possible. The lesions detected at the baseline should be followed up every 2 cycles;
- Discomforts during the treatment should be recorded and graded according to the the NCI-CTCAE, version 4.0. If necessary, develop the treatment plan with the department of oncology, and record the reasons for dose adjustment or delay, supportive treatment, and outcomes in the CRF;
- Blood pressure monitoring is performed by the patient himself/herself and recorded in a diary card. Measurement should be done at least 3 times a week during the first 2 cycles. If abnormal, the blood pressure should be monitored daily; otherwise the blood pressure is measured weekly after 2 cycles. At each visit, researchers should measure the blood pressure again. Before measurement, smoking and coffee are not permitted. Take a sitting position. The elbows should be placed at the same level as the heart, and each measurement should be taken on the same side.

4.3 Preoperative evaluation and surgical period

After completing neoadjuvant treatment, peripheral blood routine, blood biochemistry, stool routine, urine routine, 12-lead electrocardiograms, tumor markers, and coagulation function should be performed to evaluate the efficacy as well as the patients' physical condition. Without clear surgical contraindications and with the patients' consent, surgery will be performed 2–4 weeks later. If disease progresses, radiochemotherapy or salvage surgery is recommended.

- Record the results of peripheral blood routine, blood biochemistry, stool routine, urine routine, 12-lead electrocardiograms, tumor markers, and coagulation tests which are performed 7 days before and after the surgery;

- Retain pathological tissue specimens after surgery;
- Record the reasons for not surgery;
- Record operation time, blood loss, and intraoperative complications (e.g. major bleeding);
- Record postoperative complications and management;
- Record pathological type, number of lymph nodes harvested and metastasis;
- Record postoperative hospital days.

4.4 Adjuvant treatment period

Intervention group: All enrolled patients receive 6 postoperative cycles of apatinib (500 mg orally once daily on days 1 to 21) plus SOX (S-1, 40-60 mg orally twice daily on days 1 to 14; oxaliplatin, 130 mg/m2 intravenously on day 1) every 3 weeks.

- During adjuvant treatment, peripheral blood routine and blood chemistry are performed on days 1, 7 of cycle 1 and day 1 of cycles 2, 3, 4, 5, and 6;
- Physical examination, ECOG PS assessment, tumor markers, and electrocardiograms are performed on day 1 of each cycle;
- Imaging examination should be performed under the same conditions (layer thickness of scanning, use of contrast agent, etc.) every 3 cycles;
- Discomforts during the treatment should be recorded and graded according to the NCI-CTCAE, version 4.0. If necessary, develop the treatment plan with the department of oncology , and record the reasons for dose adjustment or delay, supportive treatment, and outcomes in the CRF;
- Blood pressure monitoring is performed by the patient himself/herself and recorded in the diary card. Measurement should be done at least 3 times a week during the first 2 cycles. If abnormal, the blood pressure should be monitored daily; otherwise the blood pressure is measured weekly after 2 cycles. At each visit, researchers will measure the blood pressure again. Before measurement, smoking and coffee are not permitted. Take a sitting position. The elbows should be placed at the same level as the heart, and each measurement should be taken on the same side.

4.5 Follow-up period

After the last drug administration, patients enter follow-up period. All patients are followed up with every 3 months during the first 2 years and then every 6 months beyond the third year. The status of each follow-up should be

recorded in the follow-up table.

Assessment of follow-up period: chest X-ray, whole abdominal CT, peripheral blood routine, tumor markers; gastroscopy is recommended to performed once a year.

Location and time of tumor recurrence or metastasis and cause of death should be recorded in detail.

5. Observation of Adverse Events

5.1 Definitions

Adverse Event (AE): Any adverse medical event that occurs after receiving a drug or treatment, and it is not necessarily related to treatment.

According to regulations, events that occur before and after treatment are both called AEs. Therefore, monitoring of AEs should be performed from entering the trial to the end of the visit.

Severe adverse event (SAE): Any adverse medical event requiring hospitalization, prolonged hospital stay, or causing disability, impaired work capacity, life-threatening complications/death, and congenital malformations.

5.2 Degree of Severity

Severity of AEs should be evaluated according to the NCI-CTCAE, version 4.0.

5.3 Recording and Reporting of AEs

When AEs or SAEs occur during the treatment period, the time of occurrence, clinical manifestations, management, outcomes, and their relation to the study drugs should be recorded in the CRF. Patients with abnormal laboratory tests should be followed up until the results return to normal or to pre-treatment levels. Any SAE should be recorded in the specific tables and reported to the sponsor, ethics committees, Department of Safety Supervision of CFDA, and Department of Health Administration within 24 hours.

5.4 Prevention and Treatment of Risks

Close attention should be paid to each participant during the treatment period, and management should be done in time when AEs occur.

6. Data Collection and Statistical analysis

6.1 Data Collection

The duration of data collection is from the date when ICF was signed to 30

days after the end of the last drug administration.

Each participants will receive planned visits. Specific data will be recorded at each visit.

6.2 Definitions of Analysis Sets

Full Analysis Set (FAS): included patients who receive at least one cycle of neoadjuvant treatment.

Per-protocol Set (PPS): included patients who comply with the study protocol, have good compliance, do not take pohibited drugs during the treatment, receive surgery, and complete the CRF. Analysis of the efficacy should be performed in both FAS and PPS populations.

Safety Analysis Set (SAS): included patients who receive at least one cycle of neoadjuvant treatment and have safety records.

6.2 Statistical Analysis

Baseline data are described as mean with standard deviation (SD), median, and range in case of continuous variables and as frequencies with percentages in case of categorical variables.

All statistical analyses will be conducted with SPSS software, version 22.0 (SPSS Inc., Chicago, IL, USA). A two-tailed P value less than 0.05 is considered to be statistically significant.

- Baseline characters

Calculate the mean with SD, median, maximum, and minimum of gender, ECOG score and other qualitative variables. List the frequency and percentage of age, height, weight and other quantitative variables. Categorical variables are assessed using the X2 test or Fisher’s exact test, and continuous variables are compared using Student’s t test or the Mann-Whitney U test.

- Efficacy

- Primary Efficacy Measures

Calculate the R0 resection rate with 95% confidence interval (CI).

- Secondary Efficacy Measures

Calculate the pathological/radiologic response rate with 95% CI. The Kaplan-Meier method is used to analyze overall survival (OS) and disease-free survival (DSS), and the differences are assessed with log-rank tests.

- Safety

These results mainly use description analysis. List all the AEs in this study. List the frequency and percentage of postoperative morbidity, mortality, readmission, and reoperation and the median with range of blood loss, time to food intake, and postoperative hospital days.

7. Data management

7.1 Filling and Modifying

An independent data monitoring committee is responsible for data filling and management. To ensure the accuracy, two data administrators enter the data and then proofread. If there are any questions in the CRF, the data administrators will fill in the DRQ and send a query to the researchers through the clinical monitor. The researchers should make a answer and return as soon as possible. Data modification, confirmation and entry will be done according to the response. DRQ can be sent again if necessary.

7.2 Data Lock

After data review and confirmation, the database will be locked by the principle investigator, sponsor, and statistical analyst. The locked data file can be no longer changed.

8. Quality Control and Assurance

Researchers must be experienced clinical physicians and work under the guidance of senior professionals.

The clinical ward must meet the standardized requirements to ensure that the rescue equipment is complete.

It is recommended that drug administration should be performed by professional nursing staff to ensure good compliance of participants.

Research centers must strictly follow the study protocol and enter the CRF truthfully.

The supervisor should supervise this trial according to the standard operating procedures, and confirm that all data and reports are correct and complete, all CRFs are entered correctly.

9. Ethics, regulations and administrative principles

9.1 Local regulations / Helsinki Declaration

The researchers should ensure the implementation of this study in accordance with the study protocol and in compliance with the Declaration of

Helsinki, as well as domestic and international ethical guiding principles and applicable regulatory requirements. The present study must strictly follow the "Guideline for Good Clinical Practice" ICH Tripartite Guideline (January 1997), or local law, whichever is more stringent.

9.2 Informed Consent

An unconditional prerequisite for subjects to participate in this study is his/her written informed consent. The written informed consent of subjects participating in this study must be given before study-related activities are conducted.

Therefore, before obtaining informed consent, the investigators must provide sufficient information to the subjects. In order to obtain the informed consent, the investigators will provide the information page to subjects, and the information required to comply with the applicable regulatory requirements. While providing written information, the investigators will orally inform the subjects of all the relevant circumstances of this study. In this process, the information must be fully and easily understood by non-professionals, so that they can sign the informed consent form according to their own will on the basis of their full understanding of this study.

The informed consent form must be signed and dated personally by the subjects and investigators. All subjects will be asked to sign the informed consent form to prove that they agree to participate in the study. The signed informed consent form should be kept at the research center where the investigator is located and must be properly safe kept for future review at any time during audit and inspection throughout the inspection period. Before participating in the study, the subjects should provide a copy of signed and dated informed consent form.

At any time, if important new information becomes available that may be related to the consent of the subjects, the investigators will revise the information pages and any other written information which must be submitted to the IEC/IRB for review and approval. The revised information approved will be provided to each subject participating the study. The researchers will explain the changes made to the previous version of ICF to the subjects.

9.3 Independent Ethics Committee or Institutional Review Committee

Before the study begins, all research centers should submit the study

protocol and relevant documents (ICF, CRF, and other documents that may be required) to the Independent Ethics Committee (IEC)/ Institutional Review Board (IRB). The study can only start after obtaining the approval from the IEC/IRB. Any amendment of the study protocol must be submitted to the IEC/IRB first in accordance with local laws and regulations.

9.4 Confidentiality Agreement

All materials, data (oral or written), and unpublished documents which are provided to the researchers (or any action performed by the sponsor), including this protocol and CRF, are common property of the Fujian Medical University Union Hospital and Jiangsu Hengrui Pharmaceutical Co., Ltd.

Researchers or any member of their team must not disclose such materials or data to unauthorized persons without the prior formal written consent of the sponsor.

Except the information allowed by regulations, researchers should keep all information of this study confidential, and should take all necessary steps to ensure that it will not be disclosed.

9.5 Record Keeping

Researchers should arrange for the storage of the research files until the end of the study. Additionally, researchers should adhere to specific local regulations/guidelines in record keeping.

Unless otherwise stated in the agreement, it is recommended to keep the research files for at least five years after completion or interruption of the study.

9.6 Interruption of the Study

The principal investigator can decide to discontinue this study at any time and for any reason; the decision to discontinue the study will be communicated in writing to the participating researchers and Jiangsu Hengrui Pharmaceutical Co., Ltd.

Similarly, if another researchers decide to withdraw from the study, they must notify the company in writing.

If applicable, notify the IRB health regulatory authorities in accordance with local regulations.

10. Protocol Modification

Any revisions to the protocol will be recorded in a written revision and

signed by researchers and sponsor. A signed, revised version will be attached to the protocol. A revised version should be submitted in accordance with local regulations.

11. Use of Research Results

11.1 Ownership

The use of data should be permitted by the principle investigator and Jiangsu Hengrui Pharmaceutical Co., Ltd.

The principle investigator has full access to the final data to perform appropriate academic analysis and to report of the research results.

11.2 Publicatiom

All participating researchers fully authorize the principle investigator to publish the research results for the first time. Other publication is not permitted until the first publication. Any subsequent publication (including sub-analysis) must be approved by the principle investigator and cited for the first publication of this study.

After notifying the company in advance (for internal review and comments), the principle investigator will make the final decision on the publication of any manuscript/abstract/presentation. All manuscript/abstract/presentation must be submitted to the sponsor for internal review at least 45 days before submission. Jiangsu Hengrui Pharmaceutical Co., Ltd. may require the name of the sponsor and/or the names of one or more employees of the sponsor to be listed or not listed in this publication.

Jiangsu Hengrui Pharmaceutical Co., Ltd. may delay publication or transmission for a limited period of time in order to protect the confidentiality or ownership of any information contained therein.

References

[1] Bray F, Ferlay J, Soerjomataram I, et al. Global cancer statistics 2018: GLOBOCAN estimates of incidence and mortality worldwide for 36 cancers in 185 countries. CA Cancer J Clin. 2018; 68: 394-424.

[2] Pyo Jeung Hui, Lee Hyuk, Min Byung-Hoon, et al. Long-Term Outcome of Endoscopic Resection vs. Surgery for Early Gastric Cancer: A Non-inferiority-Matched Cohort Study. Am. J. Gastroenterol. 2016; 111: 240-9.

[3] Songun I, Putter H, Kranenbarg EM, et al. Surgical treatment of gastric cancer: 15-year follow-up results of the randomised nationwide Dutch D1D2 trial. Lancet Oncol. 2010 ;11: 439–449.

[4] Cunningham D, Allum WH, Stenning SP, et al. Perioperative chemotherapy versus surgery alone for resectable gastroesophageal cancer. N Engl J Med. 2006; 355: 11–20.

[5] National Comprehensive Cancer Network: NCCN Clinical Practice Guidelines in Oncology: Gastric Cancer (version 2.2015).

[6] Okines AF, Norman AR, McCloud P, et al. Meta-analysis of the REAL-2 and ML17032 trials: evaluating capecitabine-based combination chemotherapy and infused 5-fluorouracil-based combination chemotherapy for the treatment of advanced oesophagogastric cancer. Ann Oncol. 2009; 20: 1529–1534.

[7] Yamada Y, Higuchi K, Nishikawa K, et al. Phase III study comparing oxaliplatin plus S-1 with cisplatin plus S-1 in chemotherapy-naïve patients with advanced gastric cancer. Ann Oncol. 2015; 26: 141–148.

[8] Wagner AD, Grothe W, Haerting J, et al. Chemotherapy in advanced gastric cancer: a systematic review and meta-analysis based on aggregate data, J. Clin. Oncol. 2006; 24: 2903–2909.

[9] Cunningham D, Okines AF, Ashley S, Capecitabine and oxaliplatin for advanced esophagogastric cancer. N. Engl. J. Med. 2010, 362: 858-859.

[10] Bang YJ, Van Cutsem E, et al. Trastuzumab in combination with chemotherapy versus chemotherapy alone for treatment of HER2-positive advanced gastric or gastro-oesophageal junction cancer (ToGA): a phase 3, open-label, randomised controlled trial. Lancet. 2010;376:687–97.

[11] Ohtsu A, Ajani JA, Bai YX, et al. Everolimus for previously treated advanced

gastric cancer: results of the randomized, double-blind, phase III GRANITE-1 study. J Clin Oncol. 2013; 31: 3935-3943.

[12] Okines AF, Ashley SE, Cunningham D, et al. Epirubicin, oxaliplatin, and capecitabine with or without panitumumab for advanced esophagogastric cancer: dose-finding study for the prospective multicenter, randomized, phase II/III REAL-3 trial. J Clin Oncol. 2010; 28: 3945-3950.

[13] Satoh T, Xu RH, Chung HC, et al. Lapatinib plus paclitaxel versus paclitaxel alone in the second-line treatment of HER2-amplified advanced gastric cancer in Asian populations: tyTAN-a randomized, phase III study. J Clin Oncol 2014;32:2039-2049.

[14] de Mello RA, Marques AM, Araujo A. HER2 therapies and gastric cancer: a step forward. World J. Gastroenterol. 2013; 19: 6165–6169.

[15] Janjigian YY, Werner D, Pauligk C, et al. Prognosis of metastatic gastric and gastroesophageal junction cancer by HER2 status: a European and USA International collaborative analysis. Ann. Oncol. 2012; 3: 2656–2662.

[16] Hicklin DJ, Ellis LM. Role of the vascular endothelial growth factor pathway in tumor growth and angiogenesis. J. Clin. Oncol. 2005; 23: 1011–1027.

[17] Holmes K, Roberts OL, Thomas AM, et al. Vascular endothelial growth factor receptor-2: structure, function, intracellular signalling and therapeutic inhibition. Cell. Signal. 2007; 19: 2003–2012.

[18] Tian S, Quan H, Xie C, et al. YN968D1 is a novel and selective inhibitor of vascular endothelial growth factor receptor-2 tyrosine kinase with potent activity in vitro and in vivo. Cancer Sci 2011; 102: 1374-1380.

[19] Li J, Qin SK, Xu JM, et al. Randomized, Double-Blind, Placebo-Controlled Phase III Trial of Apatinib in Patients With Chemotherapy-Refractory Advanced or Metastatic Adenocarcinoma of the Stomach or Gastroesophageal Junction. J. Clin. Oncol. 2016, 34: 1448-1454.

[20] Li W, Qin J, Sun YH, et al. Neoadjuvant chemotherapy for advanced gastric cancer: a meta-analysis. World J Gastroenterol. 2010, 16: 5621–5628.

[21] Ott K, Lordick F, Herrmann K, et al. The new credo: induction chemotherapy in locally advanced gastric cancer: consequences for surgical strategies. Gastric Cancer. 2008; 11: 1–9.

Appendix 1

Pathological Response Evaluation Criteria

Grade	Basis for Evaluation
Grade 0	No degeneration
Grade 1a	The degeneration area was less than 1/3
Grade 1b	The degeneration area was 1/3–2/3
Grade 2a	The degeneration area was 2/3–9/10
Grade 2b	The degeneration area was more than 1/3
Grade 3	No residual tumor

Appendix 2

Pathological staging was performed according to the TNM classification (AJCC, 8th edition).

- Definition of Primary Tumor (T)

Tx Primary tum or cannot be assessed

T0 No evidence of primary tumor

Tis Carcinoma in situ (intramucosal carcinoma; invasion of the lamina propria or extension into but not through the muscularis mucosa)

T1 Tumor invades the submucosa (through the muscularis mucosa but not into the muscularis propria)

T2 Tumor invades the muscularis propria

T3 Tumor invades through the muscularis propria into the subserosa or the mesoappendix

T4 Tumor invades the visceral peritoneum, including the acellular mucin or mucinous epithelium involving the serosa of the appendix or mesoappendix, and/or directly invades adjacent organs or structures

T4a Tumor invades through the visceral peritoneum, including the acellular mucin or mucinous epithelium involving the serosa of the appendix or serosa of the mesoappendix

T4b Tumor directly invades or adheres to adjacent organs or structures

- Definition of Regional Lymph Node (N)

Nx Regional lymph nodes cannot be assessed

N0 No regional lymph node metastasis

N1 One to two regional lymph nodes are positive

N2 Three to six regional lymph nodes are positive

N3 Seven or more regional lymph nodes are positive

N3a Seven to fifteen regional lymph nodes are positive

N3b Fifteen or more regional lymph nodes are positive

- Definition of Dist ant Metastasis (M)

M0 No distant metastasis

M1 Distant metastasis

GROUP	CLINICAL T	N	M
❑ 0	Tis	N0	M0
❑ IA	T1	N0	M0
❑ IB	T2	N0	M0
	T1	N1	M0
❑ IIA	T3	N0	M0
	T2	N1	M0
	T1	N2	M0
❑ IIB	T4a	N0	M0
	T3	N1	M0
	T2	N2	M0
	T1	N3	M0
❑ IIIA	T4a	N1	M0
	T3	N2	M0
	T2	N3	M0
❑ IIIB	T4b	N0	M0
	T4b	N1	M0
	T4a	N2	M0
	T3	N3	M0
❑ IIIC	T4b	N2	M0
	T4b	N3	M0
	T4a	N3	M0
❑ IV	Any T	Any N	M1

GROUP	PATHOLOGIC T	N	M
❑ 0	Tis	N0	M0
❑ IA	T1	N0	M0
❑ IB	T2	N0	M0
	T1	N1	M0
❑ IIA	T3	N0	M0
	T2	N1	M0
	T1	N2	M0
❑ IIB	T4a	N0	M0
	T3	N1	M0
	T2	N2	M0
	T1	N3	M0
❑ IIIA	T4a	N1	M0
	T3	N2	M0
	T2	N3	M0
❑ IIIB	T4b	N0	M0
	T4b	N1	M0
	T4a	N2	M0
	T3	N3	M0
❑ IIIC	T4b	N2	M0
	T4b	N3	M0
	T4a	N3	M0
❑ IV	Any T	Any N	M1

- Histologic Grade (G)

Gx Grade cannot be assessed

G1 Well differentiated

G2 Moderately differentiated

G3 Poorly differentiated

Appendix 3

Response Evaluation Criteria in Solid Tumors (RECIST) Version 1.1

Response criteria

This section provides the definitions of the criteria used to determine objective tumour response for target lesions.

- Evaluation of target lesions

Complete Response (CR): Disappearance of all target lesions. Any pathological lymph nodes (whether target or non-target) must have reduction in short axis to <10 mm.

Partial Response (PR): At least a 30% decrease in the sum of diameters of target lesions, taking as reference the baseline sum diameters.

Progressive Disease (PD): At least a 20% increase in the sum of diameters of target lesions, taking as reference the smallest sum on study (this includes the baseline sum if that is the smallest on study). In addition to the relative increase of 20%, the sum must also demonstrate an absolute increase of at least 5 mm. (Note: the appearance of one or more new lesions is also considered progression).

Stable Disease (SD): Neither sufficient shrinkage to qualify for PR nor sufficient increase to qualify for PD, taking as reference the smallest sum diameters while on study。

- Evaluation of non-target lesions

This section provides the definitions of the criteria used to determine the tumour response for the group of non-target lesions.

While some non-target lesions may actually be measurable, they need not be measured and instead should be assessed only qualitatively at the time points specified in the protocol.

Complete Response (CR): Disappearance of all non-target lesions and normalisation of tumour marker level. All lymph nodes must be non-pathological in size (<10 mm short axis).

Non-CR/Non-PD: Persistence of one or more non-target lesion(s) and/or

maintenance of tumour marker level above the normal limits.

Progressive Disease (PD): Unequivocal progression (see comments below) of existing non-target lesions. (Note: the appearance of one or more new lesions is also considered progression).

Appendix 4

ECOG PS score

According to the simplified performance status score scale developed by the ECOG, the patients' performance status can be classified into 6 levels, namely 0-5, as follows:

0: Fully active, able to carry on all pre-disease performance without restriction;

1: Restricted in physically strenuous activity but ambulatory and able to carry out work of a light or sedentary nature, e.g., light housework, office work;

2: Ambulatory and capable of all self-care but unable to carry out any work activities. Up and about more than 50% of waking hours;

3: Capable of only limited self-care, confined to bed or chair more than 50% of waking hours;

4: Completely disabled. Cannot carry on any self-care. In total, confined to bed or chair;

5: Dead.

Patients at levels 3, 4 and 5 are generally considered to be unsuitable for surgical treatment or chemotherapy.

Appendix 5

Dose Reductions and Interruption

Toxicities in the preoperative treatment are evaluated according to the National Cancer Institute-Common Terminology Criteria for Adverse Events, version 4.0. The reason for dose reductions or delay, supportive treatment performed by clinicians and outcomes will be recorded on the CRF.

1. General Considerations for Dose Adjustment

- For AEs present at baseline, the dose can be adjusted according to the change in grades. For example, if grade 1 weakness at baseline progresses to grade 2 during treatment, the dose can be adjusted as the presence of a grade 1 toxicity.
- There is no need to reduce the dose or discontinue the treatment with the presence of the toxicities which are unlikely to develop into severe events (such as hair loss, appetite changes, etc.) occur. Additionally, there is no need to reduce the dose or discontinue the treatment with the presence of anemia (non-hemolytic) because it can be improved by transfusion.
- When different grades of toxicities occur simultaneously, dose reduction should be taken according to the most severe of them.
- During treatment, if all study drugs need to be delayed or discontinued for more than six weeks, the patient will withdraw from the study. If only one drug is discontinued, the study still proceeds.
- If a patient develop a grade 3 or higher non-hematological AE, it can be discussed with the sponsor whether to reduce the dose or not.

2. Principles for Dose Adjustment and Interruption

When a grade 3/4 hematological/non-hematological AE occurs, the researcher should judge its relation to targeted agent/chemotherapy drugs: if the toxicity is related to the targeted agent, reduce the dose of apatinib to 250 mg qd; if the toxicity is related to chemotherapy drugs, reduce the dose or discontinue the treatment of Tigio or oxaliplatin. If the researcher cannot determine the relation between the toxicity and the drugs, it is recommended to discontinue apatinib first and observe for 24 hours. If the toxicity does not resolved, discontinue the chemotherapy drugs.

3. Apatinib

3.1 Dose

The initial dose is 500 mg, qd; this dose can be reduced to 250 mg, qd, or 250 mg, qod. Subsequent dose reduction is not permitted.

3.2 Principles for Dose Adjustment and Interruption

- Dose reductions are allowed in the presence of grade 3 treatment-related toxicities.
- Among non-hematological toxicities, there is no need to reduce the dose or discontinue the treatment in the presence of manageable nausea, vomiting, hair loss, fever with a defined cause (such as infection or tumor), and grade 3/4 elevated alkaline phosphatase (AKP).
- Dose reductions are allowed up to twice. Treatment interruption is allowed for no more than 14 days (cumulatively) and no more than two times in each cycle. Dose re-escalation is not permitted.
- Treatment interruption is first performed during each cycle. Then dose reduction can be performed when the subject is still intolerant.
- Special AEs: Once hypertension crisis, cerebral hemorrhage, grade 2 or higher pulmonary hemorrhage, grade 3 or higher other bleeding, arterial thrombosis, grade 4 venous thrombosis, leukoencephalopathy syndrome, or gastrointestinal perforation occurs, immediately discontinue the treatment and perform active symptomatic treatment; the subject then withdraw from the study.

Principles for Dose Adjustment of Apatinib

Classification	Grade	Regulations for Dose Adjustment
Hematologic	1-2	Maintain the original dose
	3	Discontinue the treatment until the toxicity ≤ grade 2, then continue the treatment at the original dose. If a grade 3 or higher toxicity recurs, continue the treatment after one-level dose reduction.
	4	Discontinue the treatment until the toxicity ≤ grade 2, then continue the treatment after one-level dose reduction.
Nonhematologic	1-2	Maintain the original dose
	3	Discontinue the treatment until the toxicity ≤ grade 1, then continue the treatment at the original dose. If a grade 3 or higher toxicity recurs, continue the treatment after one-level dose reduction.

	4	Discontinue the treatment until the toxicity ≤ grade 1, then continue the treatment after one-level dose reduction.

4. Tigio / oxaliplatin

Dose reductions are allowed in the presence of grade 3 hematologic or grade 2 nonhematologic toxicities. When different toxicities occur simultaneously, dose reduction or interruption should be taken according to the most severe of them.

5. Drugs Forbidden and Used with Caution

①Drugs that interfere with cytochrome P450 enzymes

Apatinib has a strong inhibitory effect on CYP3A4, CYP2C9, and CYP2C19 (IC50 <0.5 μM). CYP3A4 inducers (dexamethasone, catarrhizine, rifampin, and phenobarbital), CYP3A4 inhibitors (ketoconazole, itraconazole, erythromycin, and clarithromycin), CYP3A4 substrates (simvastatin, cyclosporine, and piperidine), other drugs metabolized by CYP3A4 (benzodiazepines, dihydropyridines, calcium-ion antagonists, and HMG-COA reductase inhibitors), CYP2C9 substrates (diclofenac, phenytoin, piroxicam, S-warfarin, and tolbutamide), and CYP2C19 substrates (diazepam, imipramine, lansoprazole, and S-mephenytoin) should be used with caution during treatment.

②Drugs that prolong QT intervals

Because apatinib is observed prolong the QT interval, drugs that prolong QT interval should be used with caution during treatment.

Antibiotics (clarithromycin, azithromycin, erythromycin, roxithromycin, metronidazole, moxifloxacin);

Antiarrhythmic drugs (quinidine, sotalol, amiodarone, propionamine, procainamide);

Antipsychotics (risperidone, fluphenazine, droperidol, haloperidol, thioridazine, pimozide, olanzapine, clozapine);

Antifungal drugs (fluconazole, ketoconazole);

Antimalarial drugs (mefloquine, chloroquine);

Antidepressant (amitriptyline, imipramine, clomipramine, dutiapine, doxepin).

③Chinese medicine and immune preparation with anti-cancer effect

SFDA-approved modern Chinese medicine preparations and

immunomodulators (such as thymosin, interferon, interleukin-2, and lentinan) are not permitted in this study.

④Bisphosphonates

In a clinical trial of bevacizumab, it was found that proteinuria was more likely to occur if disodium amidinophosphonate was used simultaneously (33.9 vs. 18.5%). Although clinically significant is rare, considering the similarity between bevacizumab and apatinib, bisphosphonates should be used with caution.

6. Drugs and treatments that can be used in combination

Patients should receive appropriate supportive therapy, including transfusions, antibiotics, etc.

All AEs should be actively treated. Symptomatic treatment can be given according to the judgment of the clinicians. All drugs used in combination should be recorded in the CRF in accordance with the regulations of the GCP.
