# Supplementary material for: Effectiveness and Safety of Apatinib Plus Chemotherapy as Neoadjuvant Treatment for Locally Advanced Gastric Cancer: A Nonrandomized Controlled Trial
Source: JAMA Netw Open. 2021 Jul 9;4(7):e2116240. doi: 10.1001/jamanetworkopen.2021.16240 (PMC8271357; doi:10.1001/jamanetworkopen.2021.16240)
Supplement: Supplement 3. — Data Sharing Statement [file jamanetwopen-e2116240-s003.pdf]

Data Sharing Statement

Lin. Effectiveness and Safety of Apatinib Plus Chemotherapy as Neoadjuvant Treatment for Locally Advanced Gastric Cancer. *JAMA Netw Open*. Published July 09, 2021. doi:10.1001/jamanetworkopen.2021.16240

Data
Data available: No

Additional Information
Explanation for why data not available: The datasets used and/or analyzed during the current study are available from the corresponding author on reasonable request.
